# Functional hydrogels for hepatocellular carcinoma: therapy, imaging, and in vitro model

**DOI:** 10.1186/s12951-024-02547-9

**Published:** 2024-07-01

**Authors:** Xiaoying Xu, Yu Liu, Yanyan Liu, Yahan Yu, Mingqi Yang, Ligong Lu, Leung Chan, Bing Liu

**Affiliations:** 1 Guangdong Provincial Key Laboratory of Tumor Interventional Diagnosis and Treatment, Zhuhai Institute of Translational Medicine, Zhuhai Clinical Medical College of Jinan University (Zhuhai People‘s Hospital), Zhuhai, 519000 Guangdong China; 2 Guangzhou First People’s Hospital, the Second Affiliated Hospital, School of Medicine, South China University of Technology, 510006 Guangzhou, China

**Keywords:** Hepatocellular carcinoma, Hydrogels, Cancer therapy, Biomedical imaging, In vitro model

## Abstract

Hepatocellular carcinoma (HCC) is among the most common malignancies worldwide and is characterized by high rates of morbidity and mortality, posing a serious threat to human health. Interventional embolization therapy is the main treatment against middle- and late-stage liver cancer, but its efficacy is limited by the performance of embolism, hence the new embolic materials have provided hope to the inoperable patients. Especially, hydrogel materials with high embolization strength, appropriate viscosity, reliable security and multifunctionality are widely used as embolic materials, and can improve the efficacy of interventional therapy. In this review, we have described the status of research on hydrogels and challenges in the field of HCC therapy. First, various preparation methods of hydrogels through different cross-linking methods are introduced, then the functions of hydrogels related to HCC are summarized, including different HCC therapies, various imaging techniques, in vitro 3D models, and the shortcomings and prospects of the proposed applications are discussed in relation to HCC. We hope that this review is informative for readers interested in multifunctional hydrogels and will help researchers develop more novel embolic materials for interventional therapy of HCC.

**Graphical Abstract:**

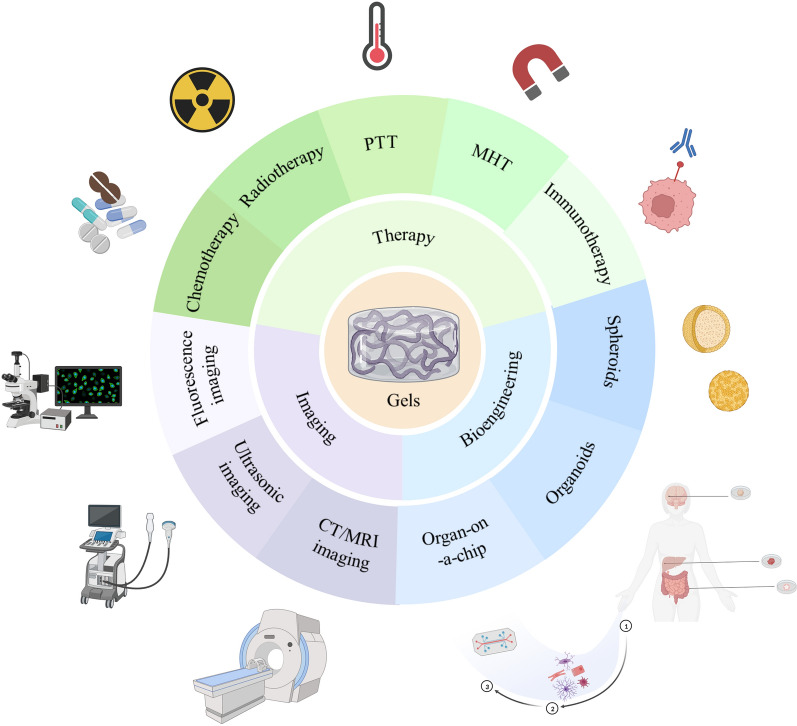

## Introduction

Hepatocellular carcinoma (HCC), accounting for approximately 90% of primary liver cancers, which is the sixth most common cancer and the third leading cause of cancer-related deaths [1, 2]. In 2020, over 900,000 people were diagnosed with liver cancer, and 800,000 people died from this disease [3]. The onset of liver cancer is a systemic, chronic, and protracted process. Well-known risk factors for liver cancer include diabetes, obesity, metabolic syndrome, chronic hepatitis B and C infection, smoking, and alcohol consumption [4]. In recent years, significant progress has been achieved in cancer treatment, and many novel imaging technologies and treatment methods have been developed. The era of comprehensive treatment has arrived, and the treatment options for liver cancer now include liver transplantation, surgical resection, ablation, chemotherapy, radiotherapy, etc. [5]. Future developments will involve accurate staging and integrated multidisciplinary care for patients with liver cancer, and tailored medical models will be created for patients, improving the overall effectiveness of liver cancer treatment. Although the numerous efficient diagnostic and therapeutic methods available to treat the condition, the prognosis for HCC is frequently unsatisfactory because the disease is typically asymptomatic in its early stages [6]. At the initial diagnosis, 80% of patients are diagnosed with middle and late stage cancer and have missed their best opportunity to be cured; thus, preventing cancer and improving the life quality of patients have become the main focus of attention [7]. At this time, transplantation, surgical resection, and radiotherapy may be infeasible, the majority of patients must receive interventional treatment. Interventional treatment for HCC includes nonvascular interventions such as local ablation, and vascular interventions such as selective hepatic artery perfusion or embolization [8]. Among these treatments, selective hepatic artery interventional therapy has the advantages of less trauma, definite curativeness and wide adaptation syndrome, so it is the most widely used in clinic. Thus, the intervention can improve the survival and quality of life of patients with unresectable HCC [9].

In the vascular interventional therapy of HCC, some transarterial embolization techniques available include bland transarterial embolization (TAE), transarterial radioembolization (TARE), and transarterial chemoembolization (TACE) [10]. Selective hepatic artery embolization can increase treatment response, lessen adverse effects, and increase patient survival rates. As additional chemical or radiation components in embolic particles are lacking, TAE is frequently referred to as "bland" embolization [11]. With this therapy, endovascular arterial occlusion induces hypoxia and subsequent death of tumor cells [12]. In a locoregional therapy called TARE, radioactive yttrium-90 (^90^Y), iodine-131 (^131^I), or rhenium-188 (^188^Re) are used to selectively inject radioactive microspheres intra-arterially and cause radiation-induced cell necrosis [13]. According to the Barcelona Clinic Liver Cancer (BCLC) staging system, TACE is the first-line treatment for patients with intermediate stage HCC, including those with large or multinodular HCC, well-preserved liver function, and no cancer-related symptoms or evidence of vascular invasion or extrahepatic spread [14, 15]. The principle of TACE is to block the hepatic artery, inhibit the blood supply of solid tumors, and achieve localized chemotherapy [16]. C-TACE, containing a chemotherapeutic agent and lipiodol, which is the recommended standard of care for the treatment of intermediate stage HCC [17, 18]. Lipoidol mixtures can cause tumor microcirculation embolism. Additionally, intratumoral retention of lipiodol can be detected in postoperative imaging, which enables the prediction of treatment responses [19]. However, an important limitation of C-TACE is the inconsistency in the technique and the treatment schedules [20]. Introducing drug-eluting bead-transarterial chemoembolization (DEB-TACE) led to more technical standardization and reduced TACE-related toxicity. The DEBs loaded with a chemotherapeutic agent (mostly doxorubicin) as a novel device can achieve tumor-specific drug delivery along with the controlled release of drugs [21]. Both C-TACE and DEB-TACE utilize the ischemic effect induced by embolic agents with deposition of therapeutic agents at a high concentration in the tumor [22]. As a result, the efficacy of tumor embolization is highly related to the performance of embolic agents [23]. Many studies have attempted to modify the structure, size, homogeneity, biocompatibility, and biodegradability of embolic agents for improving the therapeutic effects [24]. Therefore, the development of safe, effective, and multifunctional embolic materials is a crucial strategy for the development of interventional therapy against liver cancer [25].

Through continuous research and development, polymer medical materials with strong absorption capabilities, environmental sensitivity, high biocompatibility, and environmental-friendly properties have been widely prepared and researched in various fields, such as agriculture, forestry, horticulture, and biomedical fields [26]. Due to the high bioactivity and biocompatibility of polymeric medical materials, they do not cause allergic reactions in biological applications. Since the biodegradability of polymers, most biomaterials do not cause cumulative biotoxicity [27]. In addition, based on the different degradation rates of different materials, biomedical materials with different degradation rates can be prepared by adjusting the ratio of components to achieve different clinical applications [28, 29]. At present, the common embolic materials commonly used in clinical tumor intervention can be divided into solid and liquid materials [30]. Common liquid embolic agents include lipiodol and absolute ethanol, among which lipiodol is the most widely used. Lipiodol with a good fluidity can be developed under X-ray, and can selectively flow to the tumor area due to “siphon effect” [31]. However, due to the poor stability and mechanical properties, lipiodol is easy to be washed away by blood because of its low viscosity [32, 33]. On the other hand, the common solid particle embolic agents with the excellent embolization performance such as gelatin sponge, polyvinyl alcohol, drug-loaded microspheres and so on, that are often used for tumor interventional embolization. These solid particles all play the role of vascular mechanical embolization, and some of them are degradable, which can realize repeated embolization at the tumor site. The drug-loaded microspheres developed in recent years have more excellent drug loading rate, sustained release rate, targeting and other advantages [21]. However, the solid embolic agent still has some shortcomings, such as unable to embolize peripheral blood vessels, ectopic embolization and so on [34].

Hydrogels with “liquid–solid” conversion function successfully integrate the advantages of solid and liquid embolic agents and avoid its defects, and have become one of the new hotspots in the treatment of tumors in recent years. Hydrogels are an ideal embolic agent for tumor interventional therapy owing to its adjustable viscosity, mechanical properties, gelation time, injection force and embolic pressure, good biocompatibility and biodegradability, and impermeable X-ray line, as well as excellent embolization performance and intravascular diffusivity [35]. Generally, hydrogels are polymer materials that are prepared by physical or chemical cross-linking using synthetic or natural polymers, which are extremely hydrophilic gels with three-dimensional network structures [36, 37]. Novel materials based on the various environmentally responsive properties of hydrogels (pH, temperature, light, enzymes, ultrasound, etc.) can be prepared for various treatments for liver cancer, such as chemotherapy, photothermal therapy, magnetothermal therapy, photodynamic therapy, and immunotherapy, or a combination of treatments can be used to achieve better therapeutic results [38]. After imaging materials, such as tantalum powder, lipiodol, and magnetic nanoparticles, were combined with hydrogels, new hydrogels materials with imaging capabilities were prepared for visualization under imaging devices, such as X-ray, CT, MRI, or ultrasound [39–41]. Furthermore, researchers created in vitro 3D models of liver cancer based on hydrogels, such as organoids, 3D tumor spheres, and organ chips, that can replicate the spatial structure, microenvironment, and pathological functions of liver cancer in vivo, as well as other key features. As a result, the biological characteristics, pathogenesis, and metastasis mechanisms of liver cancer can be better understood for future drug screening and individualized medicine applications [42].

**Fig. 1:**
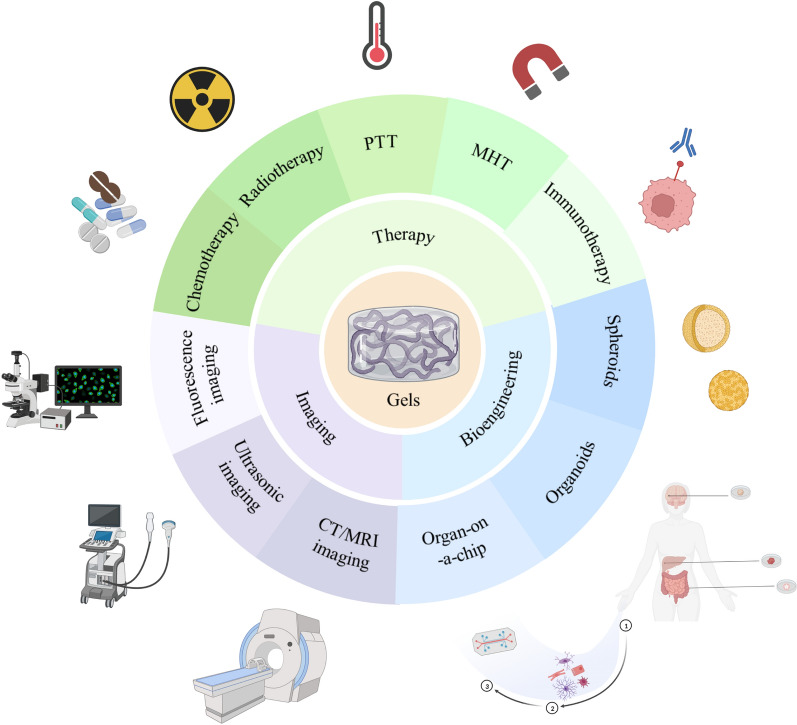
The development of research on the application of hydrogels in HCC. (Created with BioRender.com)

Therefore, based on various excellent performances of hydrogels, this review systematically presents the development of research on the application of hydrogels in HCC (Fig. 1). Firstly, the preparation of hydrogels from different cross-linking methods are introduced. Furthermore, we highlighted the therapeutic fields of chemotherapy, radiotherapy, Magnetic Hyperthermia Therapy and immunotherapy for HCC, as well as various imaging techniques and in vitro 3D models for HCC. Finally, the drawbacks and future directions of hydrogel-based multifunctional materials are summarized and discussed.

## Synthesis of hydrogels

**Fig. 2:**
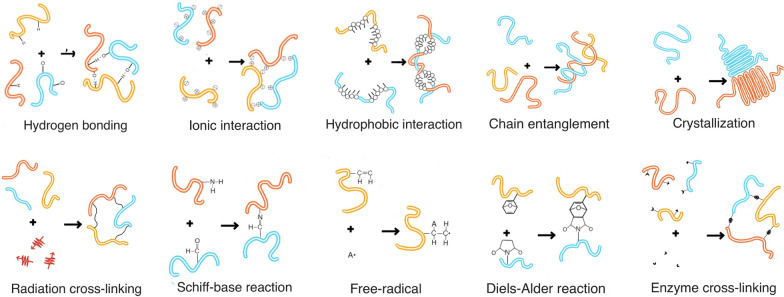
Schematic diagram of hydrogels formation by physical and chemical cross-linking

Hydrogels are a type of hydrophilic polymeric network material that can expand significantly in water [43, 44]. According to the origins of the raw materials, the substances used to create hydrogels can be divided into the following main groups: natural polymers, such as alginate, chitosan, gelatin, hyaluronic acid, fibrinogen, and peptides; and synthetic polymers, such as polyethylene glycol (PEG), polyacrylic acid (PAA), polyacrylamide (PAM), and polyvinyl alcohol (PVA). The categories of hydrogels can also be distinguished based on cross-linking techniques, such as chemical cross-linking and physical cross-linking [45] (Fig. 2). Chemically cross-linked hydrogels are built by covalent chemical bonds to form a three-dimensional network with a stable structure that is permanent and unchanging [46]. Owing to the presence of certain groups in hydrophilic polymers, such as COOH–, NH_2_–, and OH–, polymers are often covalently linked through the isocyanate-OH/NH_2_ reaction, the formation of amine-carboxylic acid, or Schiff base reactions to form the hydrogel network [44]. Physically crosslinked hydrogels are primarily crosslinked by noncovalent bonds that are reversible and dynamic [47]. When the external conditions change, synthetic hydrogels with weak mechanical properties are transferred back to the solution state. Numerous types of hydrogels exhibit distinct properties and ranges of use in practical applications. For instance, chemically cross-linked hydrogels exhibit better mechanical properties and are suitable for the preparation of slow-release medications, artificial cartilage, etc. Physically cross-linked hydrogels exhibit better water absorption properties and are suitable for the preparation of absorbent dressings, wet dressings, etc. Consequently, when determining a procedure to create hydrogels in accordance with the particular application needs and performance requirements, the proper cross-linking method must be chosen.

### Physically cross-linked hydrogels

Physical crosslinking is used to crosslink polymers into a network structure by using physical interactions between molecules to form crosslinking points. The physical interactions are mainly secondary bond valence forces, including hydrogen bonding, ionic interactions, hydrophobic interactions, chain entanglement, and crystallization [48]. The different physical interactions mainly depend on the corresponding functional groups, such as COOH–, NH_2_–, and OH–. By controlling the changes in external environmental parameters, such as temperature, pH, salt content, and other factors, the interactions between molecular chains inside the hydrogels can be influenced, and ultimately the mechanical and water absorption capabilities of the hydrogels [47]. The preparation process of physically cross-linked hydrogels is simple and does not require the use of cross-linking agents, so as to avoid the impact of harmful substances on the human body and the environment [49]. Several common physical cross-linking methods for synthetic hydrogels are described below.

#### Hydrogen bonding

Hydrogen bonding is one of the most frequently utilized non-covalent interactions in the synthesis of hydrogels. The common hydrogen bond donors mainly include carboxyl groups, amino groups, hydroxyl groups, etc. In addition, some processes can improve the performance of hydrogels, including freezing/thawing, solvent replacement, annealing, and swelling. Wang et al*.* created PVA/PVDF composite hydrogels by the annealing-swelling process to significantly improve hydrogen bonding in the hydrogels, and the hydrogen bonding between PVA/PVDF molecules was stronger than that between individual molecules [41]. The tensile mechanical capabilities of composite hydrogels also are related to different PVA/PVDF ratios. For the sample with the PVA/PVDF ratio of 7:3, the hydrogen bonding and degree of cross-linking are best, exhibited ultra-high tensile strength (6.29 MPa), elastic modulus (6.4 MPa) and stretchability (300.88%).

#### Ionic/electrostatic interactions

The ionic/electrostatic interaction between 2 molecules with opposite charges has been used in the preparation of hydrogels. For example, Wang et al. designed a hydrogel by using a high-concentration ZnCl_2 _solution and plant functional components [50]. ZnCl_2_ can form reversible ionic/electrostatic interactions with both lignin and CA, and by adjusting the relative concentration of the components to change the internal cross-linking degree. As a result, the hydrogel exhibits superior mechanical strength (tensile strength of 795.4 kPa and compressive strength of 4.1 MPa), strong/long-lasting/repeatable adhesive capability (34.8 kPa on the surface of Zn-carbon cloth in 28 days storage), good ionic conductivity (14.2 mS cm^−1^). Polysaccharide residues, natural polysaccharides negatively charged in alginate, show a strong affinity for divalent metal cations, including Ca^2+^, Sr^2+^, and Cu^2+^[51]. The interaction can quickly combine the two components to produce hydrogels without heating. Kumar *et al.* dissolved sodium alginate in Cacl_2_ and ZnCl_2_ solutions with overnight stirring to create a sodium alginate with calcium and zinc composite hydrogel (SA-CZ), which demonstrated outstanding *in vitro* and in vivo hemostatic efficiency with a wide range of wound healing potential [52].

#### Hydrophobic interactions

Hydrophobically associating hydrogels use the hydrophobic groups of amphiphilic polymers to gather inward in the aqueous phase, causing the molecular or intramolecular association of macromolecular chains to form a hydrogel network structure [53, 54]. Demott et al*.* created a triple network (TN) hydrogel with electrostatic repulsive interactions inside the first network of anionic PAMPS, hydrophobic interactions inside the second network of neutral P(NIPAAm-co-AAm), and electrostatic attraction between the third network of cationic PAPTAC and the first network [55]. After these electrostatic and hydrophobic interactions were combined, the TN hydrogel achieved an unprecedented cartilage-matching compression modulus. By adjusting the concentration of the cationic 3rd network, these TN hydrogels achieve high modulus of ≈ 1.5 to ≈ 3.5 MPa without diminishing cartilage-like water contents (≈ 80%), strengths, or toughness values.

#### Chain entanglement

Compared with small molecules, polymers with long chain structure have the property of chain entanglement. Physical entanglement occurs between linear molecular chains, resulting in chain entanglement effect. Chain entanglement is a major non-covalent reinforcement mechanism in hydrogel synthesis, which is caused by the fact that polymer chains cannot pass through each other in polymer networks. For example, Fu et al. significantly improve the stiffness of the hydrogel composed of folded elastomeric proteins by introducing chain entanglements [56]. This hydrogel combines various incompatible mechanical properties, including high toughness (250 ± 68 kJ m^−3^), high stiffness (E = 0.70 ± 0.11 MPa), and ultrahigh compressive strength (68 ± 12 MPa). This technique greatly broadens the range of mechanical properties that can be attained by protein hydrogels, showing mechanical qualities that are similar to cartilage.

#### Crystallization

Taking crystallization as the physical cross-linking point, the random curl in the solution polymer chain was induced to form a crystalline cross-linked hydrogel network [57]. By adjusting the change of temperature, ordered microcrystals can be formed in the cross-linking zone, forming a structurally stable hydrogel. Therefore, crystal crosslinking can usually be achieved by repeated freezing and thawing. Aqueous solution concentration, molecular weight, freezing temperature and time, and the number of freeze-thawing cycles will affect the properties of hydrogels in varying degrees [45]. For instance, an increase of freeze–thaw cycles of (PVA) helps to strengthen the existing crystals in the structure [58]. Increasing the initial concentration of PVA aqueous solution leads to higher crystallinity initially. The increase of molecular weight of PVA results in a higher layer thickness and broader size distribution of the crystal.

### Chemically cross-linked hydrogels

Under physiological conditions, chemically cross-linked hydrogels with covalent bonds between polymer chains often exhibit better chemical stability and mechanical capabilities than those of physical hydrogels. However, after covalent bonds are broken during deformation, they are challenging to repair. The properties of chemically cross-linked hydrogels can be adjusted by changing the type and amounts of cross-linking agents. Since organic solvents and many catalysts are needed to create chemically cross-linked hydrogels, cross-linking techniques should be continually improved and refined to address issues with biocompatibility and environmental contamination [45]. Chemical cross-linking is a three-dimensional network built by chemical reactions to form irreversible covalent bonds, which can be initiated by various reactions, such as high-energy radiation, Schiff-base reaction, free radical polymerization, Diels–Alder reaction, and enzyme cross-linking [59]. Several chemical cross-linking methods commonly used in hydrogel design are described in detail below.

#### Radiation cross-linking

Radiation cross-linking is a cross-linking process that occurs between polymer chains by γ-rays or electron beams. The benefits of radiation technology include mild reaction conditions, without initiators, pure products, and a straightforward process when compared to the crosslinking polymerization of monomers triggered by chemical initiators [60]. Hydrogels that are cross-linked and sterilized by irradiation are frequently utilized in the medical industry as burn and wound dressings. Singh et al*.* prepared a novel hydrogel from a mixture of polyvinyl alcohol (PVA), polyvinylpyrrolidone (PVP), and sterculia gum by γ-radiation to initiate cross-linking, which can be used as a dressing to deliver antimicrobial agents to wounds [61]. The increase of PVP content in the polymer will lead to a decrease of the crystallinity of the PVA segment, thus increasing the swelling of the hydrogel.

#### Schiff-base reaction

Schiff base, is the product of condensation of aldehyde group and nucleophilic amine group under physiological conditions, that has the characteristics of dynamic and reversible [62]. The hydrogel constructed by Schiff base with the advantages of simple preparation, pH responsiveness, good injectability and self-healing under physiological conditions has attracted extensive attention and research in recent years [63]. Huang et al. prepared a series of self-healing hydrogels by Schiff base with good electrical conductivity and antioxidant activity [64]. The cross-linking of these Schiff bases between the aminoglycoside antibiotics tobramycin and oxidized glucan can make tobramycin release slowly and react to pH. The storage modulus, antioxidant efficiency and Δ T of the hydrogel increased with the addition of polydopamine-coated polypyrrole nanowires (PPY@PDA NWs), and continued to increase with the increase of PPY@PDA content, enhancing the mechanical strength, antioxidant activity, and photothermal properties of the hydrogel, respectively.

#### Free radical polymerization

Free radical polymerization refers to a cross-linked network formed by chain polymerization of active radicals of unsaturated double-bond monomers or polymers [65]. Free radical polymerization is one of the common chemical cross-linking methods for the preparation of hydrogels, which has the advantages of wide application range of monomers, high reaction activity, high conversion and so on [66]. Yu et al*.* synthesized a novel variant monomer [N-acryloyl alaninamide (NAAA)] by introducing a methylene spacer between two amides in the side chain of N-acryloyl glycinamide (NAGA) and formed a supramolecular poly(N-acryloyl alanine amide) hydrogel (PNAAA) by free radical polymerization of the NAAA monomer in aqueous solution without a chemical cross-linking agent [67]. Therefore, the adjusted PNAAA hydrogel formed a transient network with good swelling and injectability. This kind of hydrogel has the ability of self-fusion and stain-resistance, and can overcome postoperative and recurrent adhesions.

#### Diels–Alder reaction

Diels–Alder reaction represents a series of highly selective cycloaddition reactions between dienes and dienophiles. As a reversible “click” reaction, side reactions and byproducts are not involved in the process, so it has become one of the important strategies for the preparation of dynamic covalent hydrogels [68]. Lueckgen et al. used Diels–Alder reaction to create a covalently crosslinked alginate hydrogel system [69]. The rheological and mechanical characteristics of this hydrogel could be altered by modifying the oxidation state of the polymer backbone, the degree of substitution of norbornene, and the ratio of norbornene to tetrazine.

#### Enzyme cross-linking

Enzymes as a catalyst can effectively trigger the chemical cross-linking of polymers containing specific functional groups to form hydrogels [70]. The enzymatic cross-linking method has the advantages of high catalytic efficiency, mild reaction conditions, and the presence of nontoxic substances [71]. Kim et al*.* established hydrogel nanomembranes on the surface of monocyte and pancreatic β-cell spheroids by cross-linking glycolic chitosan (GC) and hyaluronic acid (HA) through a streptomyces avermitilis–derived tyrosinase (SA-Ty)–mediated enzymatic reaction [72]. This kind of enzymatic cross-linking-based hydrogel nanofilm has the advantages of porous, durable and high stability. The gradually accumulated polysaccharide layer laid a foundation for islet cell transplantation.

### Physical/chemical double cross-linked hydrogels

The crosslinking mode and process of hydrogels determine its performance. Hence, innovative preparation methods are crucial for improving the performance of hydrogels. The combination of physical and chemical cross-linking is advantageous and helps unify the mechanical and biological properties of hydrogels because physically cross-linked hydrogels typically exhibit greater biocompatibility and chemically cross-linked hydrogels show better mechanical stability [73]. Yuan et al*.* synthesized AG-OD-Fe (III) hydrogels for the dynamic healing of burn wounds by using Schiff base cross-linking between catechol-modified oxidized hyaluronic acid (OD) and aminated gelatin (AG) under various –CHO/–NH2 ratios and coordination cross-linking between OD and Fe^3+^ [74]. The physical and chemical double crosslinking improved the mechanical properties and tissue adhesion of the hydrogels to varying degrees. Zhao et al*.* first generated ionic cross-linked hydrogels with strong hydrogen bonds to the surface of the matrix using Ca^2+^ and peptide-modified alginate (APD) [75]. Next, glutamine transaminases (TGases) were used to catalyze covalent heteropeptide bonds between APD and corneal ECM fibers. The results show that the double cross-linked bioadhesive hydrogels exhibit high histocompatibility, adhesion, versatility and regeneration, and can be used for large corneal defect repair.

## Function of hydrogels

### Hydrogels for HCC therapy

Compared with other materials, hydrogel presents significant advantages in the treatment of HCC. In general, the benefits of hydrogels utilized in HCC therapy can be summarized as follows: functional carrier, responsiveness, injectability, self-healing and so on. Hydrogel is a promising functional carrier because of its hydrophilicity, biocompatibility and stable 3D porous structure [76, 77]. 3D porous hydrogel can effectively protect the loaded and has a high drug loading rate. The large specific surface area gives the hydrogel great modifiability, so it is beneficial to the construction of multi-functional carrier hydrogel. Thus far, carrier hydrogels for HCC therapy can load not only small molecular drugs, protein macromolecules, but also nanovesicles and nanoparticles.

In recent years, the development of “smart” hydrogel has greatly expanded its application in the field of tumor therapy [78]. Under the external stimulation of light, heat, magnetic field or the internal environment of liver cancer, these hydrogels can undergo sol–gel transition, swelling-contraction, degradation and other response changes [79, 80]. Among them, the responsive hydrogels synthesized based on the immune and metabolic characteristics of liver cancer have made great progress. The stimulus response can realize the on-demand release of the drugs contained in the hydrogel at the tumor site and reduce the damage to normal tissues and cells [81]. Due to its shear-thinning characteristic, the injectable hydrogel can effectively protect the drugs, cells or other bioactive components embedded in the hydrogel and reduce the shear damage during injection [82, 83]. Additionally, the hydrogel exhibits the characteristics of small driving force in the process of injection and filling the irregular cavity of liver cancer [84]. Therefore, injectable hydrogels offer the benefits of minimally invasive drug delivery and local targeting, and show great medical prospects in the treatment of liver cancer. As a novel material, self-healing hydrogel has a longer lifetime than traditional hydrogel [85]. They can self-repair after cracks occur and recovery of accidental stress injury, thereby reducing the possibility of complications [86]. Therefore, the self-healing property of hydrogel is more beneficial to the development of interventional minimally invasive surgery in HCC therapy.

Hydrogels exhibit excellent biodegradability and biocompatibility, drug loading and drug controlled- release ability. A variety of new materials based on hydrogels have gained considerable attention in therapeutic applications of HCC. The materials can be used in various treatments for HCC, such as chemotherapy, radiotherapy, photothermal therapy, magnetic hyperthermia therapy and immunotherapy. A summary of the reviewed studies in this section is presented in Table 1**.**

**Table 1:** Applications of gel-based new materials in the treatment of HCC

Hydrogels	Therapy	Characteristics	Cancer cell (in vitro)	Tumor model (in vivo)	Refs.
CEC/PEGDA hydrogels	Chemotherapy	Self-healing, pH responsiveness, injectability, cytocompatibility	HepG2	–	[86]
D-PNA100	Chemotherapy	High drug-loading amount, sustained drug release, temperature sensitivity	HepG2	H22-bearing Balb/C mice	[96]
THBC hydrogel	Chemotherapy	Strong tissue adhesion, biodegradability, targeting and sustained release of drugs	—	Luc-HepG2 liver tumor-bearing SCID mice	[99]
NCTD-IRMOF-3-Gel	Chemotherapy	Inducing cell cycle arrest, obvious sustained-release effect, drug targeting delivery	Hepa1-6	–	[101]
C16-N hydrogel	Chemotherapy	Intraperitoneal depot, preferential accumulation in the liver, high cytotoxicity, sustained drug release	Bel-7402/Luc	Bel-7402/Luc- bearing Balb/c nude mice	[104]
Mucoadhesive CUR	Chemotherapy	Increase the penetration of CUR assembly inside mucus layer, inhibit growth of cancer cells at both programmed and non-programmed stages	HepG2 Huh7	–	[106]
Lipo-Dox/^188^Re-Tin colloid hydrogel	Radio-chemotherapy	Focus radio-chemotherapy locally, biodegradability, injectability, slow and stable drug release	–	BNL/luc tumor bearing Male BALB/c mice	[116]
^90^Y-RadioGel™	Radiotherapy	Physical sequestration, radiation safety, locally accurate radiation	–	VX-2 tumor-bearing rabbits	[119]
^131^I-labeled chitosan hydrogels (^131^I Chi)	Radiotherapy	Radioembolization, gamma-ray imaging, substantial liver accumulation	–	McA-RH7777-fLuc tumor-Bearing male Sprague–Dawley rats	[112]
PC10A/DOX/HAuNS nanogel	Chemo-photothermal therapy	Outstanding photothermal effect and photothermal stability, excellent biocompatibility, injectability	HepG2	HepG2 tumor-bearing male mice	[128]
Gel-SA-CuO	Photothermal and chemodynamic therapy	Glutathione depletion-induced ferroptosis, photothermia-augmented chemodynamic therapy, efficiently inhibiting postoperative tumor recurrence	H22	H22-bearing Female balb/c mice	[131]
DOX@Au-MnO-L NPs/F127 hydrogel (DAML/H)	Chemo–photothermal therapy	Injectability, stable photothermal conversion, real-time evaluation of the therapeutic effects (via MRI), long-term on-demand sustained-release	HepG2 HepG2/ADR	HepG2/ADR-bearing BALB/c mice	[132]
ICG-SF-Gel	Photothermal immunotherapy	Combining PTT and immunotherapy, strong distal antitumor effect, low side effects	H22	H22-bearing male balb/c mice	[136]
MCG	Magnetic Hyperthermia Therapy	Effective magnetic heating effect, remarkable rheological properties, high gel network stability, stimuli-responsive drug delivery, hemostasis	–	HepG2 tumor-bearing Balb/c mice, VX2 tumor rabbits	[144]
NDP-FG hydrogel	Magnetic Hyperthermia Therapy	Bioapplicable thermal-responsiveness, strong adhesion in wet conditions, high magnetic hyperthermia, biocompatibility	–	VX-2 tumor-bearing rabbits, HepG2 liver tumor-bearing mouse	[145]
ferrimagnetic silk fibroin hydrogel (FSH)	Magnetic Hyperthermia Therapy	Superior magnetocaloric killing ability, infinite penetration depth, remote hyperthermia	–	VX-2 tumor-bearing rabbits	[143]
NGC-Gels	Immunotherapy	Reverse the immunosuppression, evoke long-term immune protection, elicit strong antitumor immunity	–	Hepa1–6 tumor-bearing mice	[155]
HMPB/BLZ945/anti-SIRPα	Immunotherapy	Block MCSF-CSF-1R and CD47-SIRPα signal axes simultaneously, reduced stemness of tumor tissues, reshape the postoperative TME	HEPA1-6	Hepa1–6 tumor-bearing mice	[156]
TF-Nanogels	Immunotherapy	TAE-TVI synergistic artery embolization, inhibit tumor angiogenesis and metastasis, distinct antitumor immune response	HepG2	VX2 tumor-bearing rabbits	[157]
AuNP@PNA/DOX	Immunotherapy	X-ray angiography, favorable shear thinning effect, favorable pharmcokinetics and biocompatibility	–	VX2 tumor-bearing Rabbits, Hepa1-6 tumor-Bearing C57BL/6 mice	[158]

#### Chemotherapy

Chemotherapy is among the crucial options for numerous stages or the entire course of tumor treatment, that along with surgery and radiotherapy as the three main methods for treating cancer. Preoperative neoadjuvant chemotherapy shrinks inoperable tumors into operable lesions to reduce the risk of recurrence and metastases. Postoperative adjuvant chemotherapy primarily eliminates microscopic liver cancer lesions that remain after surgery to prevent metastases. Moreover, advanced palliative chemotherapy can lessen uncomfortable symptoms caused by advanced liver cancer, relieve pain, and delay the life span of patients [87]. Drugs used in systemic chemotherapy spread to all tissues and organs in the body through blood circulation and damage healthy cells. These side effects including nausea, vomiting, indigestion, abdominal pain, abdominal distension, constipation, and hair loss, will exacerbate any damage to the human body [88, 89]. Local chemotherapy is essential for the treatment of cancers in this situation. It is a targeted treatment in which drug concentrations are high in tumor tissues but systemic plasma drug concentrations are low. Thus, local chemotherapy can lessen the toxic side effects of chemotherapy medications and their negative effects on the body [90].

Hydrogel-based nanosystems are used to carry various functional anticancer drugs for effective, precise, and secure anticancer treatments [91, 92]. Based on the high biocompatibility, low cytotoxicity, biodegradability, high drug loading capacity, sensitive stimulus–response, and even multistimulus response, hydrogel-based drug delivery systems (DDSs) are an effective drug delivery method with significant potential in the development of HCC treatments. Due to its unique pharmacokinetic properties, DDSs stand out among many polymeric drug control release systems. These drug delivery systems show great potential for delivering anticancer medications in the context of chemotherapy for HCC, both as carriers of insoluble and unstable medications and as local drug depots. Hydrogels directly deliver medications to the desired location at high concentrations for slow and sustained release at the tumor site [93].

DOX is an anthracycline antibiotic isolated from Streptomyces and is used to treat malignant diseases, such as breast cancer, Hodgkin's lymphoma and non-Hodgkin's lymphoma, ovarian cancer, lung cancer, acute lymphoblastic leukemia, and HCC [79]. The first report on DOX for HCC was published in 1976, and it is among the earliest and most commonly used antitumor drugs for the treatment of HCC [94]. However, the high systemic toxicity of DOX causes side effects, such as bone marrow suppression, cardiotoxicity, and gastrointestinal reactions [95]. Thus, it is necessary to safely and specifically transport DOX to the lesion site. An intelligent DDS based on hydrogel can ensure that DOX is safely delivered, which greatly improves the selectivity, biocompatibility, dispersion and stability of DOX; as a result, the delivery efficiency and bioavailability of DOX are improved [79]. In a recent study, Qu et al*.* used N-carboxyethyl chitosan (CEC) and dibenzaldehyde-terminated poly (ethylene glycol) (PEGDA) to create an injectable hydrogel for DOX encapsulation via the Michael reaction [86]. This hydrogel with self-healing and pH-responsive properties achieved in situ DOX release from HCC. The release rate of DOX was faster in the acidic tumor microenvironment (pH = 5.5) than in the normal microenvironment (pH = 7.4). The cellular experiments in vitro confirmed that the hydrogel inhibited the proliferation of human HCC (HepG2).

**Fig. 3:**
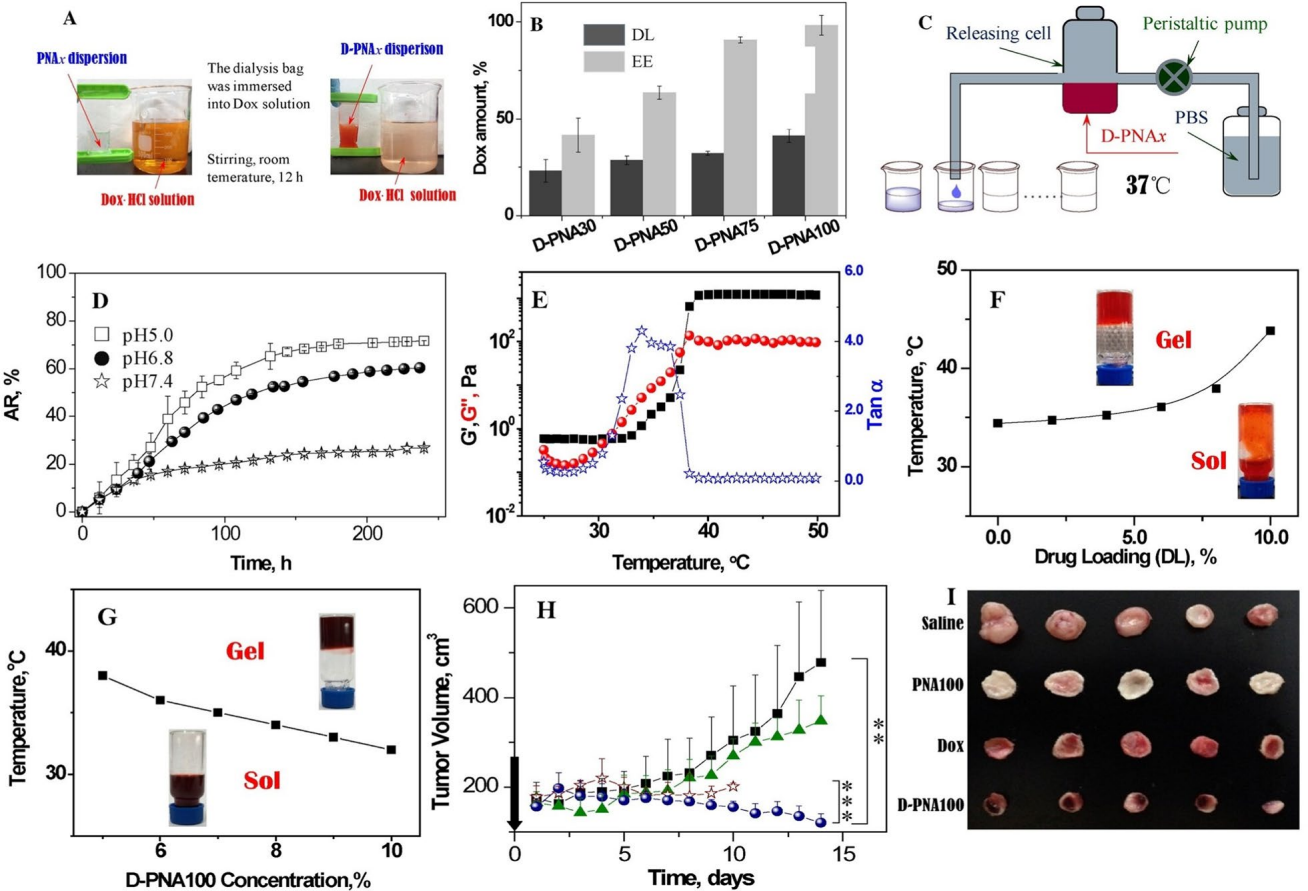
**A** The scheme of DOX loading process: DOX was transferred from the solution in a beaker into PNAx dispersions in a dialysis bag via acid–base neutralization; **B** drug-loading amount (DL) and entrapment efficacy (EE) of four D-PNAx nanoparticles with different hydrolysis degrees x; **C** The scheme of drug-eluting device for sustained release; **D** In vitro releasing profiles of D-PNA100 nanoparticles at 37 °C and various pH values. **E** the rheological curves of elastic modulus (G′), loss modulus (G″) and loss tangent (tanα) with temperature. The concentration of D-PNA100 was 10 wt.%, DL of DOX is 5.0 wt.%. (**F**) The sol–gel phase transition diagrams of DL vs temperature. PNA concentration is 10 wt.%. **G** The sol–gel phase transition diagrams of the concentration of D-PNA100 vs temperature. DL of DOX was 5.0%. **H** Tumor growth curves. **I** Gross photographs of tumor mass. Reproduced with permission from Ref. [96]. Copyright 2016, Elsevier B.V

Furthermore, hydrogels with an appropriate viscosity play a significant role in the DDS of HCC. Wan et al*.* described a temperature-sensitive triblock polymer (D-PNAx) prepared by DOX and the nanomaterial PNAx for local drug delivery in HCC (Fig. 3A) [96]. The hydrogel achieved a high drug loading and encapsulation rate and slow drug release (9.4% in 1 day; 60% in 10 days) (Fig. 3B–D). Due to the temperature sensitivity of the hydrogel (Fig. 3E–G), the loaded DOX was released sustainably in tumor tissues with a long treatment time. After intratumoral injection was performed, the tumor volume in an animal model of liver cancer was significantly reduced, demonstrating the remarkable anticancer effect of D-PNAx (Fig. 3H, I).

**Fig. 4:**
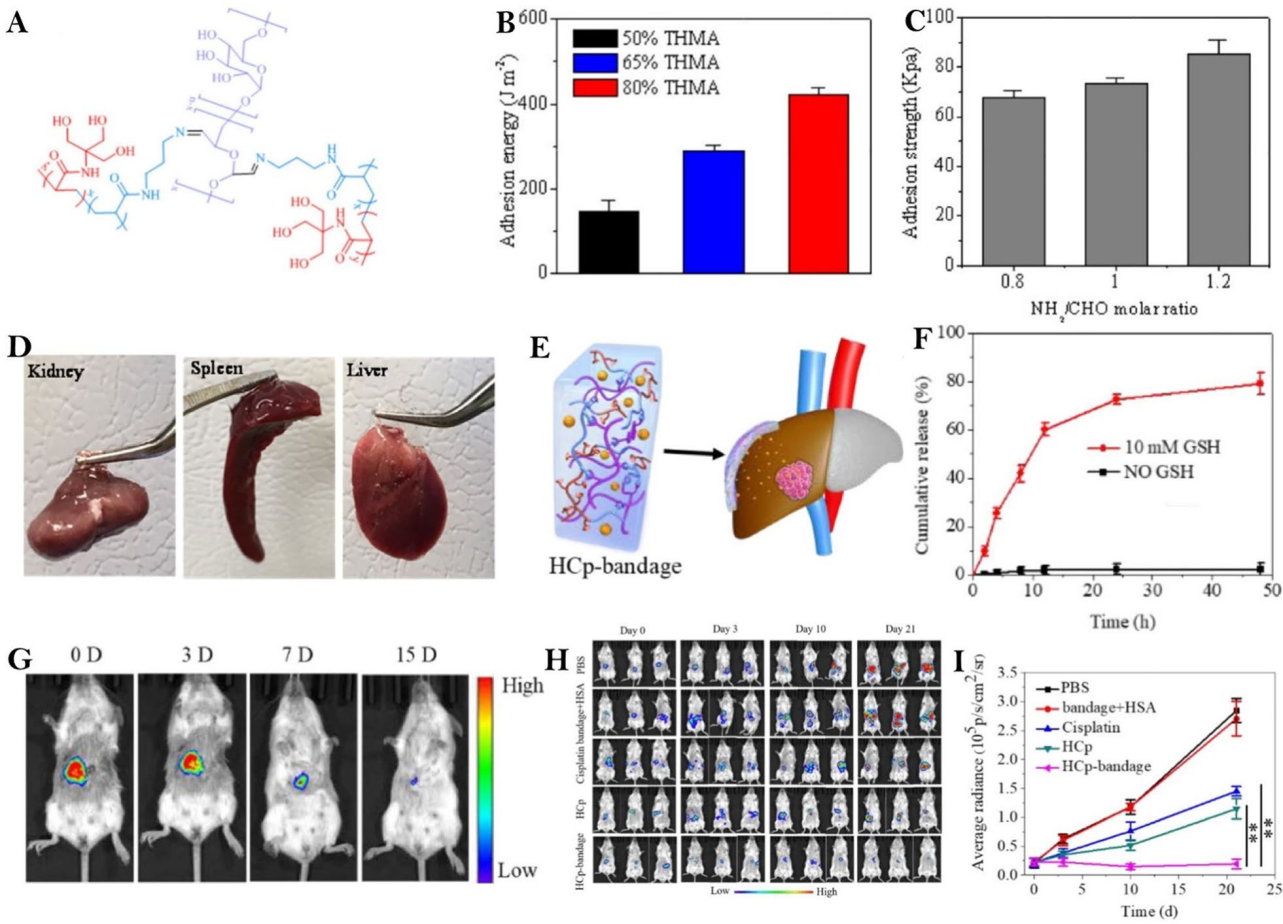
**A** A schematic of p(APMA-co-THMA) crosslinked with Dex-CHO to form biodegradable adhesive hydrogel. **B** Average adhesive energy of hydrogels with different THMA contents to glass slide as measured by the peeling test.** C** Adhesion strength of 80% THMA hydrogels with different NH_2_/CHO molar ratios. **D** Adhesion to various tissues including kidney, spleen and liver. **E** A schematic of HCp-bandage applied on the surface of orthotopic luciferase-expressing HepG2 (Luc-HepG2) liver tumor bearing liver. **F** In vivo biodegradation behavior of Cy5.5 labelled bandage after 15 days, n = 3. **G** In vitro drug release of HCp nanoparticles with and without 10 mM GSH.** H** Tumor volume changes evaluated by bioluminescence imaging in mice treated with HCpbandage, HCp, Cisplatin, blank bandage with HSA, and PBS. (n = 6; other 3 mice from each group are shown in SI). **I** Quantitative analysis of average bioluminescence levels of the mice in different treatment groups. Reproduced with permission from Ref. [99]. Copyright 2020, Wiley‐VCH GmbH

Cisplatin is known as the “penicillin of anticancer drugs” due to its widespread usage in treating cancer [97]. However, systemic chemotherapy can result in dose-related side effects, such as nephrotoxicity and neurotoxicity [98]. Chen et al*.* prepared a new type of mucoadhesive hydrogel (HCp-bandage) by coupling human serum albumin (HSA) and encapsulating cisplatin (Cp) in the hydrogel (Fig. 4A) [99]. This hydrogel matrix incorporates “triple hydrogen bonding clusters” (THBC) as a side group and strongly adheres to tissue (Fig. 4B–D). This hydrogel can firmly cling to liver tissues and perform prolonged drug release, delivering cisplatin directly to the tumor site (Fig. 4E, F). It was demonstrated that HCp bandages practically treat mice with in situ liver tumors in just three weeks without harming surrounding tissues and organs (Fig. 4G–I).

Norcantharidin (NCTD) can induce apoptosis in tumor cells by an endogenous mitochondrial signaling mechanism [100]. Li et al*.* described an NCTD-loaded metal–organic framework IRMOF-3 coated with a temperature-sensitive gel (NCTD-IRMOF-3-Gel) for efficiently transporting drugs to HCC cells [101]. The drug release curve confirmed that the hydrogel had a significantly delayed release rate, and NCTD-IRMOF-3-Gel showed strong cytotoxicity on Hepa1-6 cells with dose-dependent inhibition. The hydrogel effectively blocked the cycle of Hepa1-6 cells in the S-phase and G1/M-phase to inhibit cell proliferation. Apoptosis tests further revealed that the gel effectively induced Hepa1-6 cell apoptosis. A series of in vitro studies have shown that NCTD-IRMOF-3-Gel shows potential for stable, prolonged drug release and exhibits considerable antitumor effects, leading to an advancement in local area therapy technology for HCC.

Triptolide (TP) is a drug with a wide range of pharmacological activities, including anti-inflammatory, antitumor, and immunosuppressive actions, but it causes severe side effects and shows poor water solubility [102, 103]. Thus, Zhao et al*.* produced C16-N/T hydrogels with the peptide amphiphile C-16-GNNQQNYKD-OH and TP to utilize nanofibers [104]. This gel can be used as an intraperitoneal depot to treat HCC mice in situ by intraperitoneal injection. C16-N/T showed preferential accumulation and sustained release of tretinoin in the liver within 14 days, as well as significantly prolonged survival in mice. As a result, this hydrogel could be a good carrier for the treatment of HCC in situ.

Curcumin, a natural substance, exhibits an anti-inflammatory and antioxidant activity and could be a possible anticancer drug by inhibiting a variety of signaling pathways [105, 106]. As curcumin can couple functional groups found in polymer chains, Hanafy et al*.* exploited curcumin as a unique biological cross-linking agent to optimize a hydrogel-based drug delivery system [106]. Scientists examined the in vitro anticancer effects of curcumin on human HCC cell lines and found that curcumin inhibited the proliferation of HepG2 and Huh7 cells after 48 h of treatment (21.9 ± 3.1% and 29.8 ± 3.1%, respectively).

#### Radiotherapy

Radiotherapy for tumors includes two main types of treatment: external and internal radiotherapy [107]. External radiotherapy is still the main form of radiotherapy for liver cancer and can help patients with HCC at various stages. However, external radiotherapy sends rays to the tumor site in the body from outside the body, which usually causes more side effects than internal radiotherapy [108]. These effects include nausea, vomiting, bone marrow suppression, liver function damage, and in extreme cases, gastrointestinal bleeding [109]. Internal radiotherapy for liver cancer is treated by direct intratumoral injection or transcatheter hepatic artery radioembolization [110, 111]. The treatment causes radionuclides to accumulate in the tumor, but at a lower concentration than the tolerance dose in normal liver tissue [112]. Internal irradiation of tumors can improve the tumor control rate and reduce the side effects caused by radiotherapy. Currently, ^131^I, ^90^Y, and ^188^Re are the most frequently employed radionuclides for internal radiotherapy against liver cancer [110, 113–115]. In some investigations, these radionuclides have been used with hydrogels for radioembolization or direct intratumoral injection in HCC to improve the internal radiotherapy agent and achieve superior anticancer therapeutic results.

**Fig. 5:**
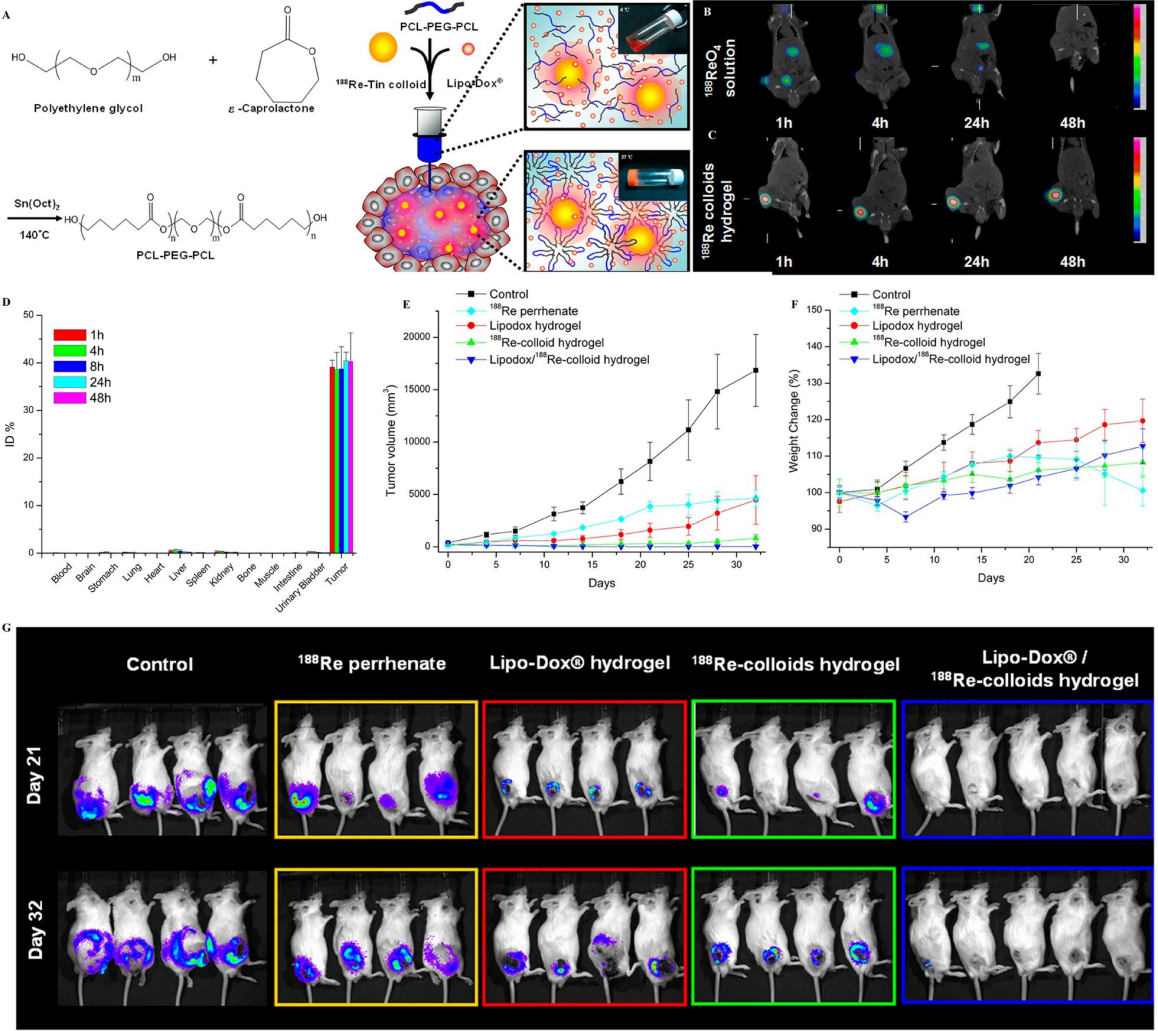
**A** Schematic depiction of the chemical structures of triblock copolymer PCL-PEG-PCL and the formation of Lipo-Dox/^188^Re-Tin colloid hydrogel at different temperatures. MicroSPECT/CT images in BALB/c mice bearing subcutaneous BNL-Luc murine liver tumor at 1, 4, 24, and 48 h after the intratumoral injection of **B** ^188^Re-perrhenate (^188^ReO4) solution and **C** Lipo-Dox/^188^Re-Tin hydrogel (equivalent to 74 MBq of ^188^Re/mouse). **D** Biodistribution of the Lipo-Dox/^188^Re-Tin colloid hydrogel at 1, 4, 24, and 48 h after intratumoral injection in BNL liver tumor-bearing BALB/c mice. **E** Tumor growth volume (mm^3^) and **F** weight change (%) of control (■), ^188^Re perrhenate (⧫), Lipo-Dox hydrogel (●), ^188^Re-Tin colloid hydrogel (▲), or Lipo-Dox/^188^Re-Tin colloid hydrogel (▼) was determined in BNL/luc tumor-bearing BALB/c mice. **G** In vivo bioluminescence imaging of BNL/luc tumor bearing BALB/c mice after various treatments on day 21 and day 32. Reproduced with permission from Ref. [116]. Copyright 2013, American Chemical Society

^188^Re emits β rays, which can have a therapeutic effect, and γ rays, which can be used for imaging to dosimetry and monitor the distribution of therapeutic agents [115]. Peng et al*.* described a temperature-sensitive hydrogel (Lipo-Dox/^188^Re -Tin) that contained the radionuclide ^188^Re and the chemotherapeutic agent Lipo-Dox for local combined radiotherapy against HCC (Fig. 5A) [116]. In vitro release curves showed that DOX could be released slowly, continuously, and stably. According to in vivo experiments, ^188^Re was mainly distributed at the intratumoral injection site and showed little retention in healthy normal tissues (Fig. 5B–D). Compared with the single treatment group, the hydrogel exhibited a significant synergistic effect with radiotherapy, and the tumor growth inhibition rate at 32 days reached 99.8% (Fig. 5E–G). Immunohistochemical analysis of the tumor showed more pronounced necrosis and vacuole formation in the hydrogel group. This confirmed that Lipo-Dox/^188^Re-Tin exhibits safe and effective synergistic effects on tumors treated with local radiotherapy.

^90^Y (Yttrium-90) is a radionuclide that can achieve localized, continuous, low-dose radiation by emitting β-rays without causing significant radiation damage to surrounding normal tissue cells [117]. The treatment can achieve precise therapeutic effects against tumors, especially for the treatment of primary liver cancer, colorectal cancer liver metastases, and other liver tumors [118]. Fisher created an injectable hydrogel called ^90^Y-RadioGelTM that contained insoluble, microscopic yttrium-phosphate particles carried by a sterile solution [119]. After the polymer solution was injected into a rabbit with a VX2 liver tumor, the solution solidified at a temperature close to body temperature (30–37 °C). Therefore, the therapeutic agent containing ^90^Y could lodge at the tumor site and deliver localized, precise β radiation. Liquid scintillation counting and microCT showed that the radiation from ^90^Y was contained in the tumor and tumor margins. Therefore, neighboring normal organs or tissues receive a modest dose of radiation dosage, or even a negligible dose. In conclusion, ^90^Y-RadioGel™ is a radiologically safe and effective therapy option for solid tumors.

^131^I emits 90% β-rays, generates shorter radiation distances and causes ionizing radiation damage and radiobiological effects on surrounding cells without affecting normal hepatic parenchymal cells [112, 120]. Hwang et al*.* developed a chitosan-based hydrogel labeled with ^131^I as a novel radioembolization device for radioembolization therapy of HCC [112]. In vivo studies showed that ^131^I-Chi was deposited in large amounts mainly in the liver, with minimal deposition in extrahepatic tissues and organs. Compared with that in the control group, tumor growth was significantly inhibited in the ^131^I-Chi-treated group. The tumor volume showed a 4.2-fold reduction in size at 28 d compared to that of the pretreatment, and extensive tumor necrosis could be observed microscopically after the tumor tissue was stained with H&E staining. Moreover, ^131^I emits 10% of the γ-rays used for imaging. These rays can be utilized to track the location and distribution of ^131^I in the body to realize the individualized treatment of liver cancer. All of these findings suggest that ^131^I-Chi has significant and unique oncological benefits.

#### Photothermal therapy

Photothermal therapy (PTT) is a new noninvasive cancer treatment technique that uses a photothermal agent (PTA) to effectively kill cancer cells [121]. As the core of PTT, the biosafety and photothermal conversion performance of PTA have a significant influence on the effectiveness of PTT [122]. Compared with conventional cancer therapies, PTT has high specificity and low toxic side effects and can easily be used multiple times [123]. In addition, deep tissue penetration, nonradiative light energy conversion, and time–space specificity are further benefits of this technology, which is currently a hot research topic in the field of cancer therapy [124–126]. Currently, a wide range of gold nanorods, hollow gold nanospheres, carbon tubes, and iron oxide are used as PTA for cancer treatment [127–130]. It is difficult to entirely eradicate remaining lesions on the margins using PTT, so it is extremely susceptible to recurrence [121]. It is anticipated that numerous PTT-based combination therapies, including chemotherapy, chemodynamic therapy, and immunotherapy, would greatly increase the overall effectiveness of tumor treatment.

Jin et al*.* synthesized a PC10A/DOX/HAuNS hydrogel based on a genetically engineered polypeptide and HAuNS that exhibited excellent photothermal effects and photothermal stability [128]. According to in vitro tests, the hydrogel can efficiently kill all HepG2 cells. After tumor-bearing mice injected with the hydrogel underwent 9 min of laser (λ = 808 nm, 2.0 W cm^−2^) irradiation, the temperature of the tumor reached 55.9 °C. Over time, the tumor volume continued to decrease, and 80% of the tumors disappeared completely. After 72 days, the recurrence rate and survival rate were 20% and 100%, respectively. In vitro and in vivo experiments have shown that tumor suppression is significantly improved through the combination of chemophotothermal therapy compared to either PTT or chemotherapy. As a result, the PC10A/DOX/HAuNS hydrogel will likely be used as a PTA to perform in vivo PTT against tumors.

**Fig. 6:**
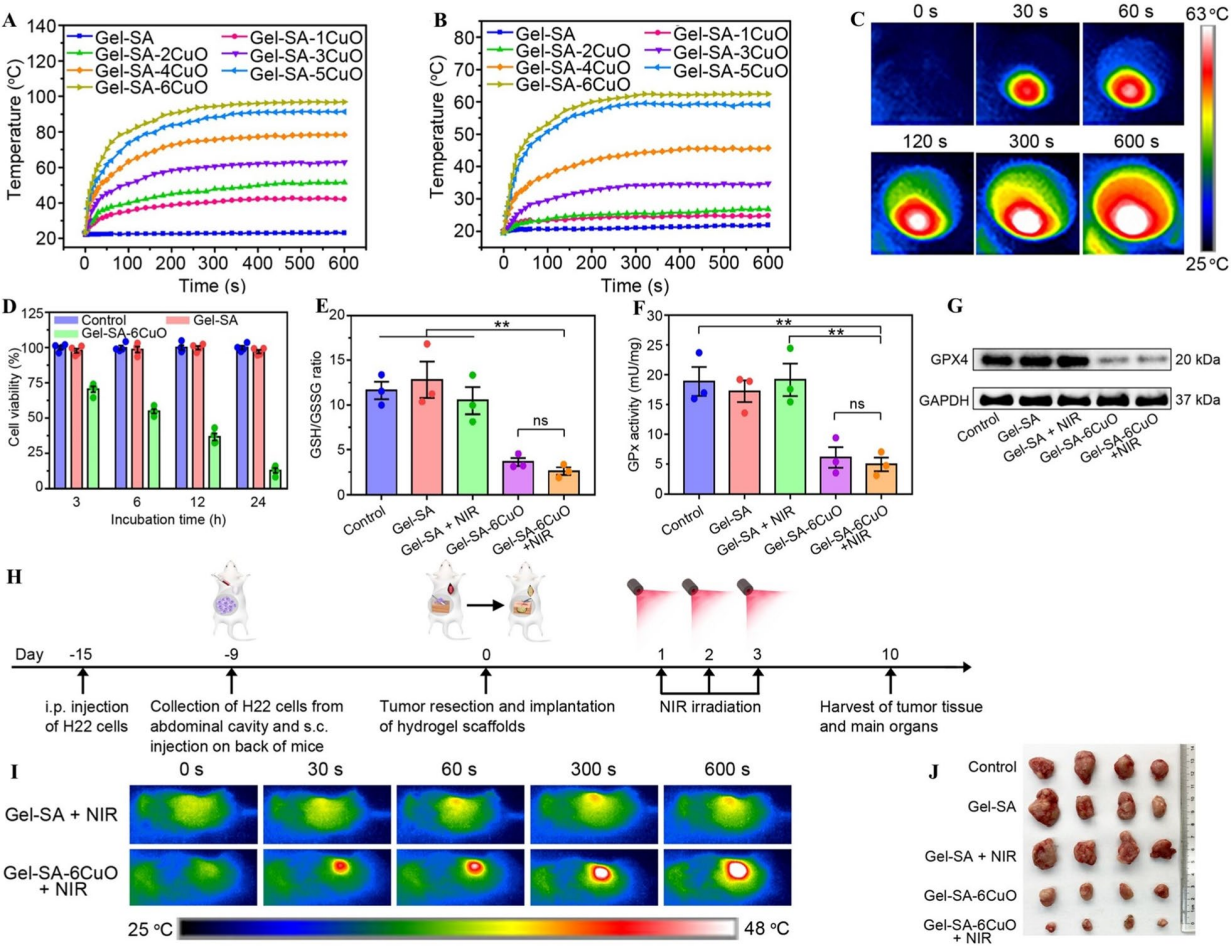
**A** Temperature changing curves of Gel-SA-CuO scaffolds exposed to NIR laser at 0.6 W/cm^2^ under dry conditions. **B** Temperature changing curves of Gel-SA-CuO scaffolds exposed to NIR laser at 1.3 W/cm^2^ under wet conditions. **C** The representative photothermal images of Gel-SA-6CuO scaffolds exposed to NIR laser at 1.3 W/cm^2^ at indicated time points under wet conditions. **D** Effect of incubation time on the viability of H22 cells treated with indicated scaffolds. **E** Measurement of GSH/GSSG ratio in H22 cells with indicated treatments. **F** GPx activity of H22 cells with various treatments. **G** The GPX4 expression of H22 cells with various treatments. **H** Schematic illustration of the use of Gel-SA-CuO hydrogel scaffolds for inhibiting postsurgical recurrence. **I** Real-time thermal images of mice treated with Gel-SA hydrogel scaffolds and Gel-SA-6CuO hydrogel scaffolds under NIR irradiation. **J** Photograph of tumors extracted from mice with different treatments on day 10. Reproduced with permission from Ref. [131]. Copyright 2022, The Author(s)

Dang et al*.* created 3D-printed hydrogel scaffolds (Gel-SA-CuO) by using gelatin, sodium alginate, and CuO nanoparticles [131]. They combined these scaffolds with photothermal and chemodynamic therapy to inhibit tumor recurrence. In addition to acting as a Cu^2+^ release reservoir to produce intracellular ROS, the CuO nanoparticles also caused GPX4 inactivation through GSH depletion-induced (Fig. 6G), which ultimately results in ferroptosis. Most significantly, it also functioned as a PTA to produce heat. When exposed to an NIR laser at 0.6 W/cm^2^ under dry conditions, Gel-SA-6CuO gradually increased to 97.00 °C (Fig. 6A). When NIR laser irradiation was at 1.3 W/cm^2^, the temperature reached 63.33 °C under wet conditions (Fig. 6B–C). These results indicated that environmental humidity had a significant influence on the photothermal properties of Gel-SA-CuO hydrogel scaffolds. After H22 cells were irradiated by a near-infrared laser for 24 h, the cell survival rate decreased to 12.802%, indicating that this scaffold exhibits a good anticancer effect (Fig. 6D–F). After NIR laser irradiation, the body temperature of the liver cancer model mice gradually increased to approximately 48 °C, and the tumor volume was dramatically reduced after therapy (Fig. 6H–J). In addition, the photothermal effect may boost the efficiency of Fenton-like reactions, thus improving their chemical kinetic properties.

Huang et al*.* created an injectable temperature-sensitive hydrogel called DOX@Au-MnO-L NPs/F127 hydrogel (DAML/H) [132]. By cooperating with chemical photothermal therapy, this hydrogel can circumvent problems caused by multidrug resistance in tumor cells, which results from long-term use of chemotherapeutic drugs. After NIR radiation was applied for 10 min in vitro and DAML/H was quickly warmed to 49.7 °C, a greater quantity of DOX was released and a maximum percentage of apoptosis was reached. The DAML/H group mice with HepG2/ADR tumors had the greatest tumor temperature (43.8 °C) following 10 min of exposure to an NIR laser (808 nm, 1 W/cm^2^). The thermosensitive hydrogel can be localized to the tumor region, increasing the photothermal conversion efficiency. A single dose can achieve on-demand and sustained-release therapy for up to 14 days. Therefore, DAML/H can be used as a promising synergistic chemotherapeutic method for multidrug-resistant liver cancer.

Indocyanine green (ICG) is a dye approved by the US Food and Drug Administration (FDA) for use in near-infrared fluorescence imaging of cancer [133]. ICG is also employed as a PTA for tumor ablation since it possesses photothermal conversion abilities [133, 134]. Ganoderma lucidum polysaccharides (GLPs) exhibit immunomodulatory and antitumor properties [135]. Based on this, Xia et al*.* reported ICG-SF-Gel for combination immunotherapy and PTT [136]. Results from in vivo and in vitro tests indicated that ICG could convert light into heat. ICG-SF-Gel could converge and accelerate ICG's photothermal action, enabling ICG to produce a stronger and faster photothermal effect. GLPs increased the photothermal efficacy of ICG, and the combination of the two significantly prolonged the survival of mice. HE staining and immunohistochemical staining of treated mouse tumors indicated that the tumors in the ICG-SF-Gel group exhibited much more extensive necrosis and a considerable drop in Ki-67 expression compared to those in the other groups. Therefore, this hydrogel can improve the abscopal effect of PTT on HCC mice through immunomodulation, anti-proliferation, pro-apoptosis, and anti-angiogenesis.

#### Magnetic hyperthermia therapy

Magnetic hyperthermia therapy (MHT) is a remote and noninvasive treatment for cancer [137]. Magnetic nanoparticles (typically paramagnetic iron oxide nanoparticles) are exposed to alternating magnetic fields to vibrate and cause local temperature to increase, achieving a MHT effect against tumors [137, 138]. MHT achieves a good effect and penetration depth, so it is frequently used to treat clinical tumors, such as glioblastoma, prostate cancer, brain cancer, and other malignant tumors [139–141]. Notably, MHT mediated by magnetic nanomaterials is beneficial because tissue penetration is practically unlimited [137, 142]. Therefore, MHT can be used to treat deep cancer, especially liver cancer [142, 143]. MHT has attracted widespread attention in the field of nanomedicine because of its ability to deeply penetrate tissues and selectively kill tumors as well as its high biological safety [142].

Chen et al*.* reported an injectable magnetic colloidal gel (MCG) that is assembled from magnetic montmorillonite (MMT) and amphoteric gelatin nanoparticles (GNPs) [144]. After MCG_DOX_ was exposed to an alternating magnetic field (AMF), which exhibits a thermal effect that can kill cancer cells, the temperature of MCG_DOX_ rose to approximately 53 °C within 10 min. In addition to improving mobility during magneto-thermal stimulation, MCG_DOX_ contributed to quicker drug release. Compared with that of the control group, drug release in the MCG_DOX_ group increased by approximately double. The MCG_DOX_ group of HepG2 tumor-bearing mice exposed to an AMF (H = 30 kA/m, f = 312 kHz) showed an increase in tumor region temperature to 42 °C in just 12 min and the lowest recurrence rate (0%) after 14 days. This result illustrated that MCG exhibited superior magnetic heating capabilities and tumor suppressive effects. MCG offered further minimally invasive treatments for rabbits with VX2 tumors and was successfully applied with ultrasound-guided interventional MHT to prevent postoperative recurrence. The optimized MCG achieved a multifunctional combination of hemostasis, stimulation-responsive drug delivery, and magnetic thermotherapy, enabling synergistic treatment of postoperative HCC in the prevention of tumor recurrence.

Yan et al*.* constructed an in situ formed magnetic hydrogel (NDP-FG) by using a triblock polymer matrix (NIPAM-co-DOPA, NDP) and reduced graphene oxide nanosheets decorated with iron oxide nanoparticles (Fe_3_O_4_@rGO, FG) [145]. Under the effect of an AMF, the temperature of the aqueous NDP-FG dispersion quickly rose from 20 to 49 °C within 10 min. Due to the incorporation of magnetic FG nanosheets, the composite hydrogel achieved effective magnetothermal therapy and sufficiently killed liver cancer cells but only caused negligible damage to normal cells. In addition to enabling magnetothermal therapy for HCC, the multifunctional hybrid hydrogel permitted intraoperative hemostasis and transarterial embolization for HCC. Therefore, these two methods show good application prospects in the treatment of HCC.

**Fig. 7:**
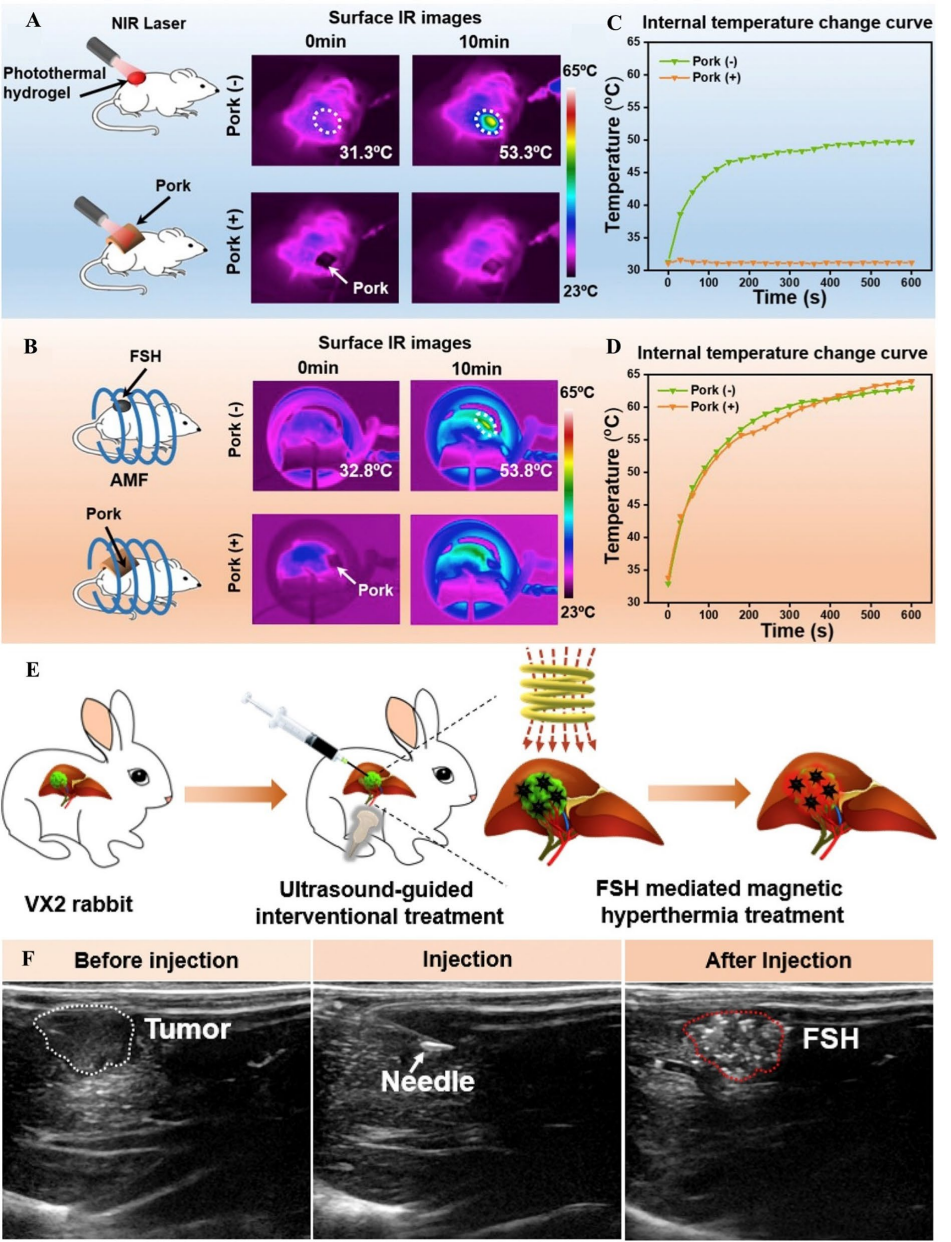
**A** Scheme illustration and infrared thermal image of mice with subcutaneous injection of ASH when exposed to NIR irradiation, without or with the coverage of pork tissue on the injection site. **B** Scheme illustration and infrared thermal image of mice with subcutaneous injection of FSH when exposed to AMF, without or with the coverage of pork tissue on the injection site. **C**, **D** The relative temperature change curves of Au-silk fibroin hydrogel (ASH) in (**A**) and FSH in (**B**) recorded by a thermoelectric couple. The concentration of Fe_3_O_4 _in FSH was 2 mg Fe/ml. **E** The diagram of ultrasound guided percutaneous puncture combined with magnetocaloric therapy in the treatment of deep tumor. The FSH was injected into VX2 tumor by percutaneous puncture under ultrasonographic guidance, and then the rabbits received MHT. **F** Ultrasound guided injection of FSH. White dash line and red dash line indicated VX2 tumor and FSH in tumor, respectively. Reproduced with permission from Ref. [143]. Copyright 2020, Elsevier Ltd

Silk fibroin (SF) is a natural polymeric fibrous protein obtained from silkworm cocoons and exhibits several advantages, including water solubility, the capacity for structural modifications, good biocompatibility and biodegradability, etc. [146, 147]. Composite hydrogels based on SF have become a research hotspot for new antitumor treatment methods that can treat glioblastoma, breast cancer, liver cancer, stomach cancer, and other tumors [143, 148–150]. Qian et al*.* described a ferrimagnetic silk fibroin hydrogel (FSH) synthesized from SF and iron oxide nanocubes (IONCs) [143]. The obtained FSH retained the shear thinning behavior and could be injected by a syringe. A typical sol–gel transition of the IONCs and silk fibroin hybrid solution is observed before and after ultrasonication. One of the outstanding features of FSH is its ability to provide effective magnetothermal therapy to tumors in deeper locations. Under AMF, this ability was demonstrated in a subcutaneously implanted tumor model in BALB/c mice after fresh pork tissue was covered (Fig. 7A–D) and in an orthotopic transplantation liver tumor in rabbits (Fig. 7E, F). However, unlike photothermal therapeutic agents, the final temperature could be remotely controlled and FSH achieved a better penetration depth. Therefore, FSH was beneficial for the treatment of some deeper tumors, especially liver tumors.

#### Immunotherapy

The majority of patients with HCC have a background of liver damage or inflammation because HCC is a typical inflammation-associated malignant illness [151, 152]. The immune microenvironment of HCC is composed of immunosuppressive cells (e.g., myeloid-derived immunosuppressive cells, tumor-associated macrophages, and regulatory T cells), immune effector cells (e.g., CD8^+^ cytotoxic T cells and effector CD4^+^ T cells), the cytokine milieu (e.g., IFN-γ, TGF-β, TNF, etc.), and tumor cell-intrinsic signaling pathways (e.g., MCSF-CSF-1R and CD47-SIRPα) [153]. These cells interact and play a key role in the development of HCC and response to antitumor therapy. Immunotherapy can improve the immune function of patients by triggering the antitumor immune response in the body and preventing tumors from forming and progressing or it can directly enable the death of tumor cells by activating immune effector cells in an organism [154]. Various immunotherapeutic gels that promote tumor vascular normalization, improve the immunosuppressive microenvironment of tumors and activate the antitumor immune response are becoming a key area in research on cc for liver cancer.

Zhao et al*.* prepared NGC-Gel by integrating Hepa1-6-specific neoantigens, CpGODN, and the STING agonist 2,3-cyclic-GMP-AMP (cGAMP) via cross-linking and combining with TIM-3 antibody [155]. NGC-Gel significantly promoted CD8^+^ T-cell infiltration and activated long-lasting effector T cells, thus preventing tumor cell proliferation and prolonging the survival of patients. Synergistic immunotherapy with NGC-Gel and TIM-3 blockers induced a significant reduction in regulatory T cells in tumor tissues, overexpression of IFN-γ and lL-12p70, increased proportions of IFN-γ^+^CD8^+^T cells and 41BB^+^CD8^+^T cells, and a reduction in Foxp3^+^CD25^+^T cells. As a result, HCC and its distant metastases were effectively eliminated in situ, and the overall survival of mice was significantly prolonged by more than 180 days.

**Fig. 8:**
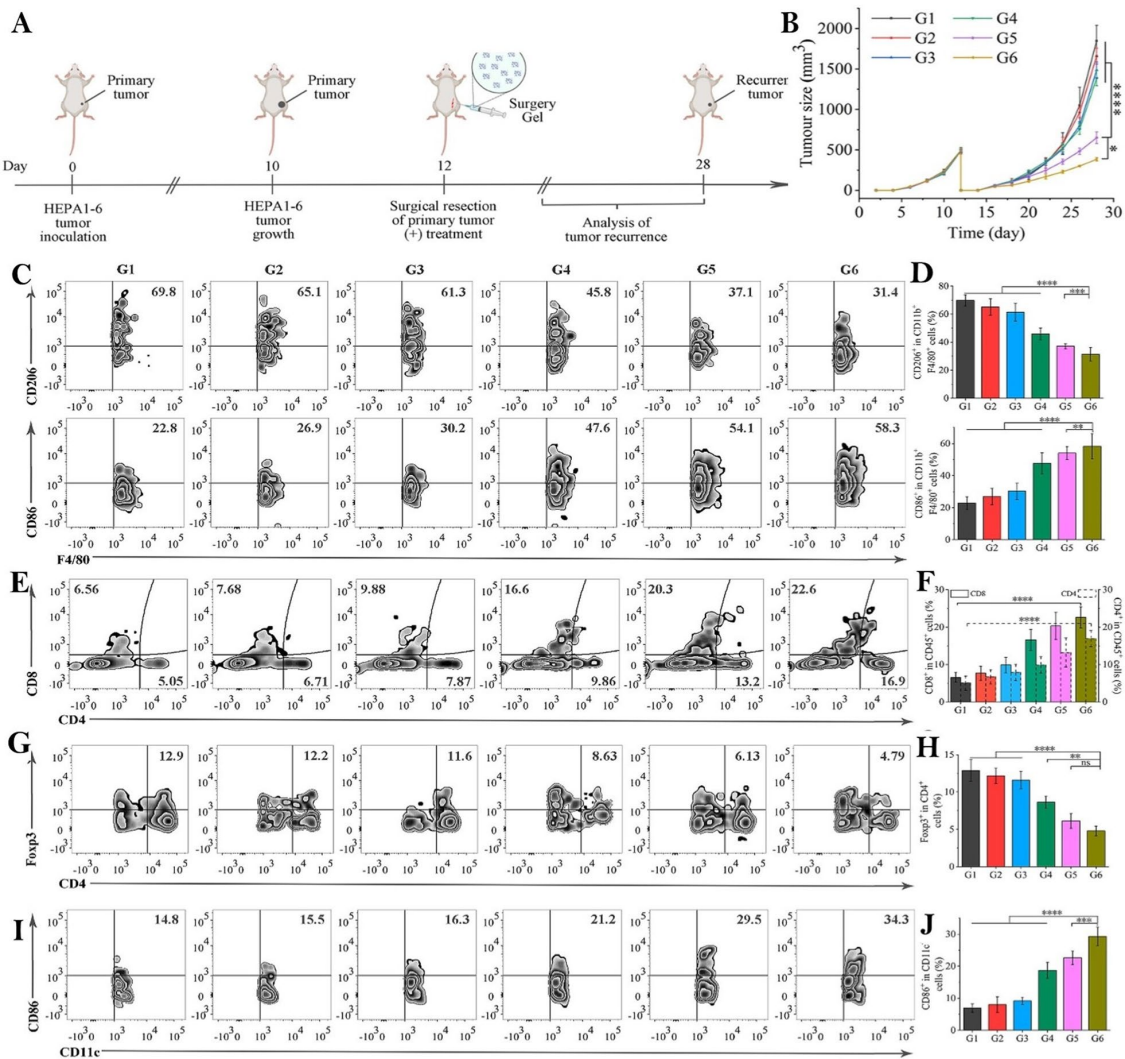
**A** Schematic illustration of HMPB/BLZ945/anti-SIRPα@ATRA@fibrin therapy in a mouse model of incomplete tumor resection. **B** Average tumor growth kinetics in different groups. **C**, **D** Flow cytometric analysis of M2-like macrophages (F4/80^+^CD206^+^) and M1-like macrophages (F4/80^+^CD86^+^) in tumor tissue. **E**, **F** Flow cytometric analysis of CD8^+^ and CD4^+^ T cells in tumor tissue. **G**, **H** Flow cytometric analysis of Tregs (CD4^+^Foxp3^+^) in tumor tissue. **I**, **J** Flow cytometric analysis of dendritic cells (DCs) in tumor tissue. For **C**–**J**, G1 was control group and G2 to G6 were treated with HMPB@fibrin, HMPB/anti-SIRPα@fibrin, HMPB/BLZ945@fibrin, HMPB/BLZ945/anti-SIRPα@fibrin, HMPB/BLZ945/anti-SIRPα@ATRA@fibrin, respectively. All the data were presented as mean ± S.D. (n = 3). Statistical significance was calculated by ordinary-one-way ANOVA and Tukey’s test. ns, not significant, **p < 0.01, ***p < 0.001, ****p < 0.0001. Reproduced with permission from Ref [156]. Copyright 2022 Elsevier B.V

Hu et al*.* created HMPB/BLZ945/anti-SIRPα as a multifunctional immunotherapeutic in situ fibrin gel for addressing challenged caused by postoperative recurrence of HCC (Fig. 8A, B) [156]. This immunotherapeutic gel can release BLZ945 and anti-SIRP at the surgical site of HCC, allowing the MCSF-CSF-1R and CD47-SIRP signaling pathways to be blocked simultaneously. Eventually, tumor-associated macrophages (TAMs) polarized or were reprogrammed from the M2 phenotype to the M1 phenotype, restoring the macrophage's ability to phagocytize cancer cells (Fig. 8C–D). In addition, all-trans retinoic acid (ATRA) can induce the differentiation of tumor stem cells (CSCs) and increase their susceptibility to immunotherapy. The multifunctional immunotherapeutic gel successfully activated the antitumor immune response and almost completely inhibited HCC recurrence (Fig. 8E–J).

**Fig. 9:**
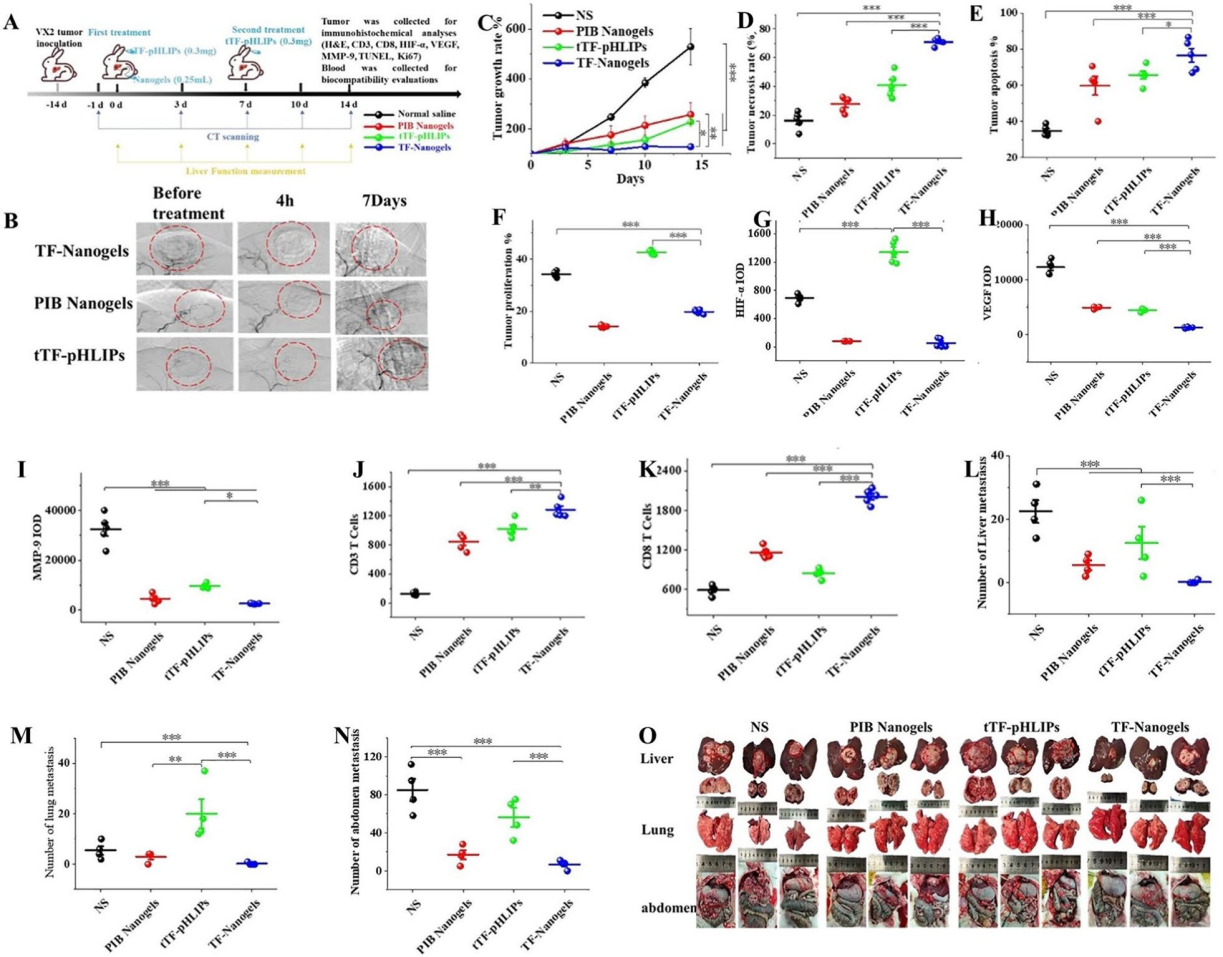
**A** Experimental design. **B** DSA image of VX2 transplant tumor in a rabbit at different periods. **C** Tumor growth rate, **D** Tumor necrosis rate, **E** Tumor apoptosis rate, and **F** Tumor proliferation rate of different groups. **G**–**K** IOD of the slices of the above staining. The number of metastasis nodules in liver (**L**), lung (**M**) abdominal cavity (**N**) at 19 days post-treatments. **O** Photos of metastasis in liver, lung and abdominal cavity. The scale bar is 100 µm. *p < 0.05, **p < 0.01, ***p < 0.001. Reproduced with permission from Ref. [157]. Copyright 2021 Elsevier B.V

Shi et al*.* prepared TF-Nanogels by compounding tTF-pHLIPs with poly(N-isopropyl acrylamide-co-butyl methacrylate) (PIB) nanogels [157]. The nanogel was delivered to the feeding artery of HCC via superselective interventional catheters to effectively inhibit tumor recurrence (Fig. 9A–F). The immunohistochemical analysis of the VX2 tumor-bearing rabbit tumor showed that TF-Nanogels exhibited a significant antitumor immune response. Compared with the other groups, the TF-Nanogel group efficiently promoted the infiltration of lymphocytes, such as CD3^+^ and CD8^+^ T cells, into tumors (Fig. 9G–K). As a result, the tumor microenvironment was remodeled and the antitumor metastasis effect of TF-Nanogels was further improved (Fig. 9L–O).

Liu et al*.* prepared AuNP@PNA/DOX by modifying gold nanoparticle AuNPs onto the temperature-sensitive block polymer PNA [158]. The hydrogel was a multifunctional embolic agent that encapsulated DOX and was used to improve postoperative tumor immune microenvironment suppression. Immunohistochemical staining of tumor tissues showed that the expression levels of HIF-1α, VEGF, and MMP-9 decreased, which improved the hypoxic tumor microenvironment and inhibited tumor metastasis. The resulting tumor-associated antigens can also promote the infiltration of immune cells, such as CD3^+^ and CD8^+^ T cells, increasing the activation and release of related cytokines, such as TNF-α and IFN-γ. This can promote the transformation of tumors from immunosuppression to immune activation, providing a favorable tumor microenvironment for immunotherapy treatments against HCC.

### Hydrogels for imaging of HCC

Importantly, HCC is the only solid tumor among all tumors that does not require a pathological diagnosis and can be diagnosed by imaging [159]. However, a possible exception occurs when the imaging presentation is atypical, as the addition of other ancillary investigations may be necessary [160]. Imaging can be used to determine the type, location, size, extrahepatic invasion, and metastasis of the tumor to provide a basis for early diagnosis, accurate staging, and treatment planning of HCC [18]. In addition, a variety of imaging techniques can be used in treatment or adjuvant therapy, such as interventional embolization guided by X-ray imaging systems or the use of imaging materials as therapeutic agents for intraoperative localization and postoperative monitoring. Several imaging modalities, including X-ray, CT, MRI, ultrasound, and fluorescence imaging, have been widely employed in the clinical workup of HCC and, after decades of continuous progress, play a crucial role in treating this illness [159]. A growing area of research is blending imaging agents, such as tantalum powder, lipiodol, and metal nanoparticles, for X-ray or CT imaging; SONAZOID and Definity microbubble contrast agents for ultrasound imaging; magnetic nanoparticles for MRI imaging; and NIR-II fluorescent dyes for fluorescence imaging. By combining these reagents with gels and other materials, multifunctional reagents can be created for diagnosis, drug delivery, and intraoperative and postoperative monitoring. These reagents show great potential for the treatment of HCC. Examples of hydrogels mentioned in this section for HCC imaging is presented in Table 2.

**Table 2:** Examples of hydrogels mentioned in this section for HCC imaging

Hydrogels	Imaging methods	Imaging materials	characteristics	Cancer cell (in vitro)	Tumor model (in vivo)	Refs.
Hydrogel-enveloped microrobots	Ultrasound imaging, x-ray imaging, MRI	SONAZOID, iodinated contrast agent, Fe_3_O_4_ nanoparticles	Magnetic actuation, real-time imaging (ultrasound, x-ray and MRI), targeted delivery, biocompatibility, biodegradability	Hep3B	N1S1-bearing Sprague–Dawley rats	[41]
ICG-loaded C/GP hydrogels	Fluorescence imaging	ICG	Injectability, high viscosity, high compactness, adequate gelation rate, intraoperative fluorescence imaging, biodegradability	–	Isolated bovine liver	[167]
hPNA nanogels	X-ray imaging	Lipiodol	Temperature sensitive, high thermodynamic stability, high yield stress in gel phase, favorable flowability and shearing-thinning property in sol phase	–	VX2-tumor-bearing rabbits	[40]
TALE	X-Ray Fluoroscopy Imaging	Tantalum ethoxide	Deep penetration, controllable phase transition, excellent biocompatibility	HepG2	HCC-grafted Sprague Dawley rats	[39]
AuNP@PNA/DOX	X-ray imaging	AuNPs	Temperature responsive sol–gel phase transition, favorable shear thinning effect, X-ray angiography, favorable pharmacokinetics and biocompatibility		VX2 tumor-bearing Rabbits, Hepa1-6 tumor-bearing C57BL/6 mice	[158]

**Fig. 10:**
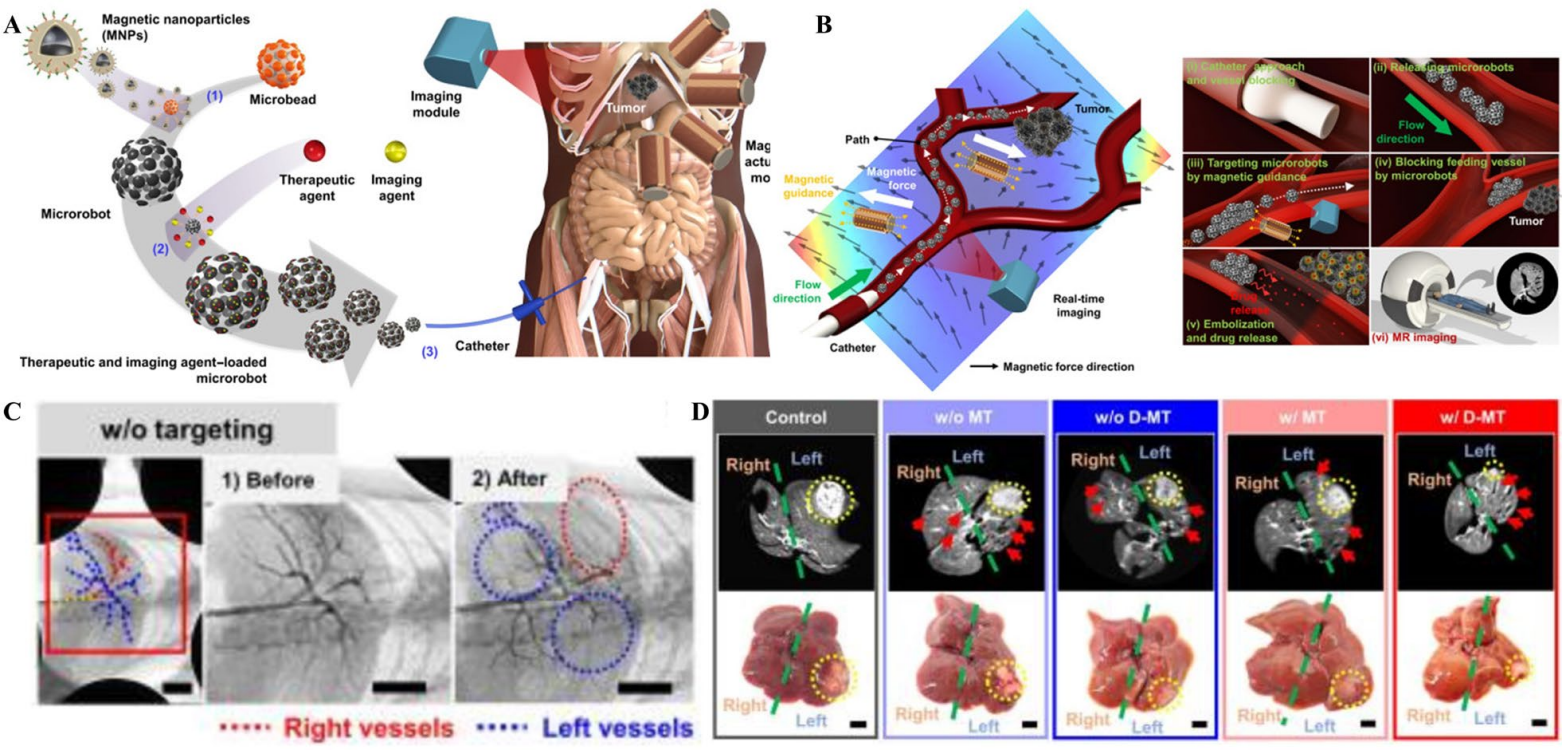
**A** Schematic illustration of fabrication and catheter delivery of microrobots containing therapeutic and imaging agents. (1) MNPs attached on the microbead. (2) Loading of therapeutic and imaging agents on microrobots. (3) Delivery of microrobots through catheter connected to the liver vessel around the tumor. **B** Schematic illustration of the in vivo tumor treatment procedure using the microrobot system. **C** X-ray angiography images of the portal vein in the RML, LL, and LML of rat liver before and after microrobot delivery. **D**MR and optical images of livers of rats in all five groups. The five groups are the control, without microrobot targeting (w/o MT), without DOX-loaded microrobot targeting (w/o D-MT), with microrobot targeting (w/MT), and with DOX-loaded microrobot targeting (w/ D-MT) groups. Red arrows on MR images indicate microrobots delivered to the rat liver. Yellow circles indicate liver tumors in rats. Scale bars, 5 mm. Reproduced with permission from Ref. [41]. Copyright 2022, AAAS

Microrobots enable precise route tracking and are frequently employed in biomedical applications for drug delivery, diagnostics, local biopsies, etc. [161]. Through microrobots, in vivo movement can be imaged via MRI, CT, and fluorescence imaging [162]. In practical clinical work, embolization of tumors must meet not only postembolization imaging requirements but is also particularly important to achieve accurate real-time imaging intraoperatively. Go et al*.* prepared a multifunctional medical microrobot system by loading the drugs DOX and 5-FU onto gelatin beads and MNPs (Fig. 10A) [41]. By loading microbubbles and iodinated X-ray contrast agents for ultrasound and X-ray imaging, intraoperative real-time visualization was realized (Fig. 10B). While brief, the X-ray imaging period is much longer than its targeting time, which is sufficient to meet its localization requirements (Fig. 10C). The MNPs, a component of the microrobot, can also be used for postoperative MRI imaging; as a result, the biodistribution of MNPs and tumor outcome can be monitored in a noninvasive manner. With both MRI imaging or gross observation, the tumor volume of liver cancer in the microrobot group was significantly smaller than that in the control group (Fig. 10D). The multifunctional microrobot can perform magnetic actuation, drug delivery, real-time visualization, and postoperative imaging, greatly enhancing the accuracy and safety of interventional surgery.

ICG is the most commonly used fluorescent dye reagent in clinics. Excited by 750–810 nm light, ICG can emit NIR at a peak wavelength of 840 nm and causes virtually no side effects within the safe dose range [163]. ICG can provide clear, contrasting fluorescence images of liver cancer and paraneoplastic tissue aggregates in contrast to normal tissue [164]. The role of ICG-based NIR fluorescence imaging in the detection, visualization, and navigation of HCC has been reported in many studies [165, 166]. Salis et al*.* developed ICG-loaded hydrogels with the potential for intraoperative fluorescence imaging and local therapy of HCC as embolic agents [167]. A strong affinity was observed between ICG and the hydrogel due to their electrostatic interaction. The results showed that the hydrogel can maintain ICG well when loaded with ICG and does not impact the degradation rate. The ICG dye-loaded hydrogel solidified rapidly at body temperature after injection, so the material can mark areas of liver tumors. The injection site of the hydrogel was visualized intraoperatively by an NIR fluorescence imaging system to realize NIR fluorescence imaging and local treatment of HCC.

**Fig. 11:**
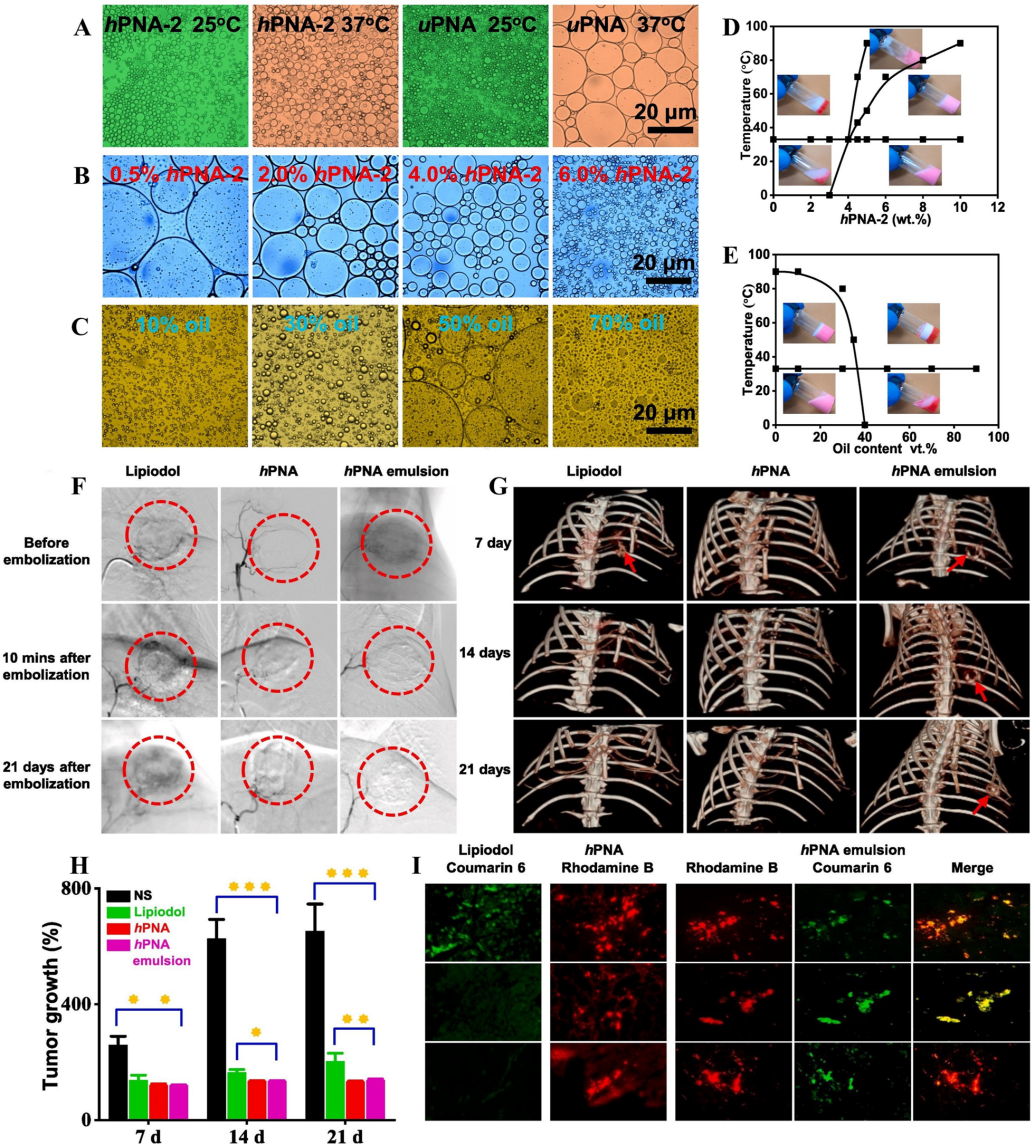
**A** Morphology of Pickering emulsion emulsified by hPNA-2 and uPNA at 25 °C and 37 °C (6 wt% nanogel dispersions as aqueous phase, 30% oil content). **B** Emulsifying effect of different hPNA-2 content (hPNA-2 nanogel dispersions as aqueous phase, 30% oil content). **C** Emulsion morphology of different oil content (6 wt% hPNA-2 dispersions as aqueous phase). **D** Phase diagram of hPNA-2 content—Temperature (30% oil content, 200 μg/mL nile red was dissolved in oil). **E** Phase diagram of oil content—Temperature (6 wt% hPNA-2 dispersions as aqueous phase, 200 μg/mL nile red was dissolved in oil). The pH of all samples is 4.0. **F** DSA images of tumors before and post-embolization on 10 min and 21 days. Iohexol (350 mg I/mL) was used as a contrast agent. The red dashed circle marks the position of the tumors. **G** 3D reconstruction CT imaging of tumors after embolization with the above three embolic agents for 7, 14 and 21 days. The red arrow shows the location of the embolized tumors. **H** Tumor growth rate at 7 and 14 days after embolization. **I** fluorescence sections of tumors at 7, 14 and 21 days after embolization (hPNA nanogels were labelled with rhodamine B, coumarin 6 was dissolved in Lipiodol, × 200). *p < 0.05, **p < 0.01, and ***p < 0.001. Reproduced with permission from Ref. [40]. Copyright 2021, Elsevier B.V

Lipiodol is impermeable to X-rays and can specifically accumulate in HCC tissues; thus, lipiodol can be used as a carrier of various chemotherapeutic drugs and has been utilized generally in the clinical embolization of hepatocytes [31, 168]. To improve the embolization strength and stability of Lipiodol in TACE therapy of HCC patients, Li et al*.* fabricated a temperature-responsive Pickering gel emulsion with highly dispersive stability (Fig. 11A–E) [40]. The hPNA-stabilized Pickering emulsion can cause rapid and long-term occlusion of tumor arteries. In rabbit renal artery embolization, a CT scan showed that the vessels were completely occluded for 56 days. The TAE antitumor efficacy in VX2 tumor-bearing rabbits indicated that the hPNA-stabilized Pickering emulsion achieved complete embolization with no demulsification (Fig. 11F). Because of the X-ray impermeability of the hPNA-stabilized Pickering emulsion, the signal intensity of CT images of embolic tumors remained unchanged during the observation period. The results showed that the hPNA-stabilized Pickering emulsion helped improve long-term embolization to tumor arteries and exhibited a significant antitumor effect (Fig. 11G–I). These results indicated that the gel emulsion may be developed as a novel embolic agent for TAE therapy to achieve radiographic imaging and long-term embolization of HCC.

Tantalum exhibits a strong radiopaque property due to its high atomic number, so it is often mixed with other embolic materials to prepare radiopaque embolic agents for visualization under X-ray [169–171]. Ko et al*.* developed tantalum alkoxide liquid embolic (TALE) based on tantalum ethoxide [39]. This is a novel multifunctional embolic agent that can be observed under X-ray fluoroscopy. Although the contrast exhibited by the contrast agent decreased with increasing oleyl alcohol content in tantalum ethoxide, the improvement in contrast was sufficiently high under X-ray fluoroscopy to meet clinicians' needs for intraoperative detection of embolic agents. TALE is a liquid embolic agent that can achieve deeper penetration. In contrast to other forms of embolic agents, TALE can successfully provide guidance in real time and monitor the tumor response at early stages after treatment under the X-ray imaging system. T_75_O_25_ was synthesized by mixing the angiogenesis inhibitor sorafenib (SOF) with TALE for TACE in rat HCC transplants. The results showed that almost all SOF accumulated in the tumor and achieved remarkable therapeutic effects.

Gold nanoparticles (AuNPs) are the most widely studied precious metal nanoparticles, with multiple surface functions and unique localized surface plasmon resonance [172]. AuNPs have a wide range of clinical applications, such as antibacterial and biosensor applications, targeted drug delivery, and cancer therapy [172, 173]. In addition, AuNPs can be used as an effective X-ray imaging agent to monitor efficacy during tumor treatment. Liu et al*.* prepared a multifunctional embolic agent (AuNP@PNA/DOX) based on AuNPs [158]. The embolic agent generated concentration-dependent X-ray attenuation that displayed high-density shadowing on CT scan images. Due to its strong and excellent X-ray shielding effect, arterial embolism can be visualized for up to 90 days. AuNP@PNA/DOX considerably inhibited tumor cell proliferation and metastasis in the VX2 rabbit liver cancer model. According to these studies, AuNP@PNA/DOX has a promising future in the clinic as a multifunctional vascular imaging and embolization agent.

### Hydrogels for in vitro model of HCC

Liver cancer is among the most common malignant tumors worldwide, is characterized by high morbidity and mortality, and is a serious threat to human life and health [3]. Before therapeutic approaches for liver cancer can be effectively applied to patients, the therapies must undergo a series of repeated and lengthy preclinical cellular and animal studies. Although animal models of liver cancer are the "gold standard" for preclinical testing, there are limitations in terms of ethics, cost, and trial periods [174]. More complex and physiologically relevant in vitro models constructed from patient-derived HCC cell lines can assist in treatment validation by providing additional humanized preclinical models. The method can circumvent the limitations of in vivo animal tumor models and is expected to be among the alternative technologies that replace animal tumor models. The most commonly used HCC cell line for in vitro HCC models is HepG2 [175]. Other HCC cells used for in vitro studies include Huh7, HCC-LM3, SNU-449, Bel-7405, HEP3B, Hep G2.2.15, SNU-182, and QGY-7701 [42]. These cell lines share similar biological characteristics to primary HCC cells and have unrestricted passage, thus providing an ideal in vitro model for HCC research.

Conventional 2D tumor cell culture is currently the main method for in vitro experiments, with the advantages of low-cost and high-throughput [42]. However, since flat tumor cells are adapted to artificial in vitro culture conditions, it is difficult to retain the intrinsic heterogeneity and phenotypic characteristics of the original tumor [176]. It is also difficult to reproduce key features of tumor spatial structure, microenvironment, and intercellular interactions [177]. Therefore, 2D cell culture methods and the data collected based on them may be misleading and unpredictable for in vivo applications [177]. HCCs are in a 3D environment in vivo, so increasing research in recent years has favored in vitro 3D hepatocellular carcinoma cell culture. With this method, the microenvironmental parameters of cells (temperature, chemical gradient, oxygen, pH, etc.) can be more easily controlled and monitored. Then, a suitable microenvironment is provided for individual cells to maintain their normal 3D shape and structure, successfully simulating the construction of tumor structure and function in vivo [176]. Ultimately, the heterogeneity of tumor cells and the impact of their microenvironment will be studied to achieve multicellular coculture that traditional two-dimensional culture cannot achieve.

Bionic in vitro 3D tumor models, such as 3D tumor spheroids, organoids, and organs-on-a-chip, can mimic the key features of in vivo tumors to explore the biological properties, pathogenesis, and metastasis mechanisms of HCC [178–180]. These models have become among the hottest research topics today and are widely used in disease research, new drug development, and regenerative medicine [181]. Tumors grow in an extracellular matrix (ECM) composed of proteins, glycoproteins, etc. [182]. Among the currently available biomaterials, polymeric hydrogels are a very common and appropriate choice for mimicking natural ECM and guide a variety of cellular behaviors [183]. The material has a high water content, relatively well-defined composition, mostly tunable physicochemical properties, and good biocompatibility [184]. Numerous hydrogel-based 3D models of HCC have been created in vitro. These models have accelerated the development of 3D in vitro cell culture techniques for HCC and led to more in-depth research on the cell‒cell and cell-extracellular matrix interactions in HCC. The models also help researchers study the effects caused by tissue-specific stiffness, oxygen, nutrients, and metabolic waste gradients on the action of HCC [176]. Examples of hydrogels mentioned in this section for HCC imaging is presented in Table 3.

**Table 3:** Examples of hydrogels mentioned in this section for in vitro 3D model of HCC

3D tumor model (In vitro)	Model cells	Characteristics	Application	Refs
Tumor spheroids	HepG2	High cell fixation, high tumor formation rate, tight cell arrangement, high volumetric 3D aggregates, cytoskeleton rearrangement	In vivo xenografted tumor model	[185]
tumor spheroids	HepG2	Controlled stiffness,controlled intercellular organization, phenotype, and angiogenic activity	Study the effect of matrix stiffness on malignant level of 3D tumor sphere	[188]
Tumor spheroids	HepG2 Hep3B	High drug resistance, low drug penetration, mimic the tumor microenvironment	High-throughput drug screening	[191]
Organoids	14 different HCC-PDX lines	Retain viability, proliferative capacity, genomic profile, gene expression and intra-tumoral heterogeneity	Drug testing	[193]
Organ-on-a-chip	HepG2	Ordered micro-nano structure, uniformity of cell sphere, adequate supply of nutrients high cell survival rate, reproducible and accurate drug screening	High-throughput drug screening	[194]

Chen et al*.* conducted a study on the utilization of 3D hydrogels for human hepatocellular carcinoma cell culture systems in vitro and established an in vivo xenografted tumor model [185]. HepG2 cells grew in 3D within the hydrogel, resulting in the formation of more aggregated cell clusters and longer culture times. In 3D cell culture, the cytoskeleton of HepG2 cells changed, and the cytoplasmic actin microfilaments rearranged, which showed structural similarity with tumor tissue in vivo. HepG2 tended to aggregate into more multicellular spheroids in 3D cell culture, and the cellular characteristics, such as cell morphology and interaction between cell membranes, actin cytoskeleton, and nucleus, were different from those in 2D culture.

The stiffness of hydrogels can affect cell survival, migration, adhesion, and tissue-specific functions [186]. Some studies have reported that the mechanical rigidity of hydrogels is mainly controlled by collagen concentration and plays a key role in regulating the malignancy of cancer cells attached to the colloidal surface [187]. Liang et al*.* encapsulated HepG2 cells in a hydrogel with controlled rigidity and limited permeability variation to produce a cell-instructive hydrogel [188]. This modified matrix rigidity could control cell adhesion to the stroma and neighboring cells, ultimately producing 3D tumor spheres with various degrees of malignancy. The spheres were characterized by controlled cell proliferation, metabolite detoxification, and tumor vascular growth. Compared to cells with E_0_ = 4.0 kPA, the tumor cells in gels with E_0_ = 0.7 kPA proliferated faster, formed larger tumor spheres, and expressed higher levels of β-1 integrin and VEGF on the 9th day.

The differentiation, proliferation, migration, tumor microenvironment, as well as the signaling mechanisms between cells and the extracellular matrix, can be better replicated in tumor spheres created through 3D culture [176]. Therefore, 3D tumor spheroids are gradually replacing planar cultured tumor cells, becoming among the most popular models in in vitro tumor research for tumor invasion studies and high-throughput screening (HTS) of drugs [189, 190]. Lee et al*.* developed a 3D aggregated spheroid model (ASM) after optimizing the 3D cell culture method [191]. In the HTS test in vitro, the model exhibited higher resistance to six different anticancer drugs than the conventional spheroid model (CSM) and a 2D cell culture model. Western blot analysis of cells in various cultures revealed that the epithelial cell marker E-cadherin in ASM was more highly expressed, suggesting that the epithelial features of HCC cells were more effectively preserved in ASM. The expression of proteins associated with cancer cell proliferation (p-AKT, p-Erk) and drug resistance markers (Fibronectin, ZO-1, Occludin) was also significantly higher in ASM patients than in controls. Therefore, ASM can better simulate the tumor microenvironment of human solid tumors, reduce the cost of experiments, increase the throughput of drug analysis and testing, and facilitate large-scale drug screening.

**Fig. 12:**
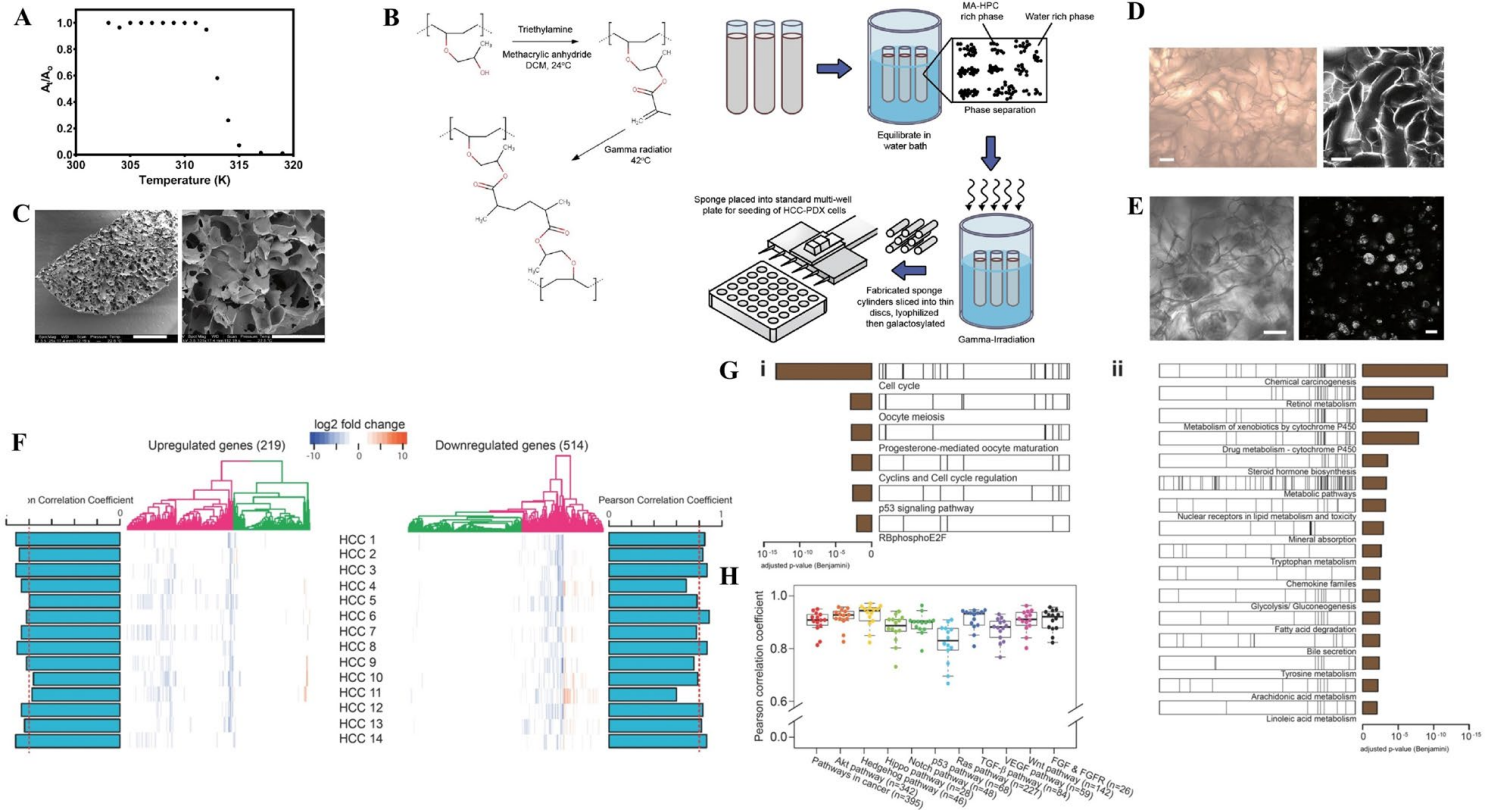
**A** Normalized UV absorption as MA-HPC undergoes thermal-induced phase separation with increasing temperature. Aqueous MA-HPC exhibits low critical solution temperature transition, from isotropic solutions at room temperature to metastable colloidal systems upon heating. Phase transition occurs at the precipitous decrease in transmittance with increasing temperature. **B** Reaction scheme of MA-HPC and sponge fabrication process. **C** Scanning electron micrographs of MA-HPC sponge at low (left, scale bar = 1 mm) and high (right, scale bar = 300μm) magnifications, highlighting macroporosity of sponge. **D** Brightfield image of MA-HPC image when dry (left) and hydrated (right, stained with propidium iodide). **E** Brightfield (left) and phalloidin-stained (right) images of HCC-PDX cells cultured in MA-HPC sponge. Scale bars = 100 μm. **F** Heatmaps of fold change differences (PDX vs 3D PDX) in expression levels (FPKM) of known up-regulated and down-regulated HCC genes between PDX and 3D PDX. Each row in the heatmap represents the expression profile of individual HCC-PDX lines (HCC 1–14). Each column represents a gene. Blue lines in the heatmap indicate lower gene expression in PDX as compared to 3D PDX and red lines indicate higher gene expression in PDX as compared to 3D PDX. Globally, the heatmaps for both down-regulated and up-regulated genes are largely white, indicating similarity in gene expression profile between PDX and 3D PDX. This was quantified using Pearson correlation coefficient as indicated by the blue bars on the left of the heatmaps. **G** i. Differentially expressed genes (up-regulated in HCC) between PDX and 3D PDX are significantly enriched in cell cycle and p53 signaling pathways amongst others (brown bars). ii. Differentially expressed genes (down-regulated in HCC) between PDX and 3D PDX are significantly enriched in metabolism-related pathways amongst others (brown bars). Black lines represent individual genes in these enriched pathways, where their position along the white bar corresponds to their position in the heatmap in (**F**). **H** Beeswarm plot of Pearson correlation coefficient between PDX and 3D PDX for nine cancer-related pathways and overall pathways in cancer. Each dot represents a matched in vitro-in vivo HCC-PDX pair. Reproduced with permission from Ref. [193]. Copyright 2018, Elsevier Ltd

The organoid model derived from tumor tissue formation is a "mini" organ with a 3D structure, which can better preserve the relevant characteristics of tumors [192]. The model provides a reliable reference to help researchers determine the pathogenesis of cancer and develop drugs to prevent and treat cancer. Fong et al*.* synthesized a 3D macroporous sponge system composed of hydroxypropyl cellulose (HPC) and methacrylate (MA) (Fig. 12A, B) [193]. This hydrogel exhibited a highly macroporous structure with a wide pore size of 80-180 μm, a porosity of 94.8%, and mechanical properties up to 9.7 kPa (Fig. 12C–E). The key features, such as tumor cell proliferative activity, genomic profiles, and tumor heterogeneity, of the organoids formed by inoculating different HCC-PDX lines onto the sponge were well preserved, providing robust culture conditions for HCC-PDX organoids in vitro (Fig. 12F–H). Additionally, HCC-PDX cells cultured in sponges showed good sensitivity in drug assays, which is beneficial for future HCC drug development.

Among the multiple methods used to treat liver cancer with poor prognosis and high mortality, simple and practical medication therapy is the main treatment method. Organ-on-a-chip technologies that can be employed for preclinical drug screening and patient treatment response prediction are emerging for individualized treatment coupled with tumor heterogeneity. Zhu et al*.* generated a hierarchical hydrogel system with ordered micro-nano structures by using the template replication method for liver cancer cell culture, drug screening, and organ-on-a-chip construction [194]. Due to the presence of ordered micro-nano structures, the cell spheres cultured in the hydrogel were more uniform in size and achieved a higher long-term survival rate than those cultured on 2D surfaces or simple microstructures. The hierarchical hydrogel system was integrated into a multichannel concentration gradient microfluidic chip and a functional liver cancer-on-a-chip was prepared. This chip achieved high-throughput drug screening with good repeatability and high accuracy. Due to these features, the hierarchical hydrogel system and its integrated liver cancer chip are an excellent platform for personalized medication screening.

## Conclusion and prospects

This review focuses on hydrogels that were modified and compounded in different ways and their significant role in the treatment of liver cancer with chemotherapy, radiotherapy, MHT, PTT, and immunotherapy. In addition, the combination of hydrogels with materials impervious to X-rays is reviewed, as these hydrogels can perform imaging and better monitor treatment effects against liver cancer. Finally, it is introduced that the biofunctional materials provided by hydrogels offer new opportunities for research in the field of organoids, spheroids, and organs-on-a-chip for liver cancer. Compared to previous technology, the application of these hydrogels to 3D models of liver cancer in vitro generates a preclinical test that is more scientifically rational, providing more accurate and improved next-step guidance for the treatment of HCC. In the past few decades, researchers have been modifying and compounding hydrogels to further diversify their functions. Hydrogels exhibit more biocompatibility, higher water absorption and water retention, higher elasticity and toughness, and biodegradability.

Although research on hydrogels for oncology applications has been active and achieved good results in recent decades, there are still some drawbacks. First, the experimental results obtained from novel materials based on hydrogels remain a problem in clinical applications. The in vitro 3D model of HCC mentioned in the paper can preserve key features, such as spatial structure, tumor heterogeneity, and cellular interactions, and is more accurate than the test data obtained through 2D tumor cell culture and animal tumor models. However, due to the immaturity of related technologies, these bionic in vitro models cannot fully encapsulate the complexity of the tumor microenvironment in vivo. Even if the models are becoming more similar to real human tumors, differences are inevitable. Therefore, experimental results can only be close to results obtained in vivo and cannot be directly transformed into practical clinical applications. Second, hydrogel materials with flexible stimulus–response properties under external or internal physiological stimuli, such as pH, temperature, light, enzymes, and magnetic fields, are increasingly being investigated for the local treatment of tumors. However, this property may affect the chemical or physical stability of a hydrogel in human tumors, leading to failure or decomposition of the material and reducing its therapeutic or other effects. Finally, modifying hydrogels to prepare more functionalized hydrogel materials is a sophisticated technique, so further research is needed to achieve large-scale production applications. In summary, hydrogel materials have been greatly progressed in tumor treatment, imaging, and in vitro bionic models, but shortcomings still remain. In future research, we can continue to study the modification of hydrogel-based material composites in detail, develop novel hydrogels with more intelligent and multifunctional properties, and examine a broader application field.

## Data Availability

Not applicable.

## References

[1] Chakraborty E, Sarkar D (2022). Emerging therapies for hepatocellular carcinoma (HCC). Cancers.

[2] Bertuccio P, Turati F, Carioli G, Rodriguez T, La Vecchia C, Malvezzi M, Negri E (2017). Global trends and predictions in hepatocellular carcinoma mortality. J Hepatol.

[3] Sung H, Ferlay J, Siegel RL, Laversanne M, Soerjomataram I, Jemal A, Bray F (2021). Global cancer statistics 2020: GLOBOCAN estimates of incidence and mortality worldwide for 36 cancers in 185 countries. CA Cancer J Clin.

[4] McGlynn KA, Petrick JL, El-Serag HB (2021). Epidemiology of hepatocellular carcinoma. Hepatology.

[5] Chidambaranathan-Reghupaty S, Fisher PB, Sarkar D, Sarkar D, Fisher PB (2021). Hepatocellular carcinoma (HCC): epidemiology, etiology and molecular classification. Mechanisms and therapy of liver cancer.

[6] Zheng Z, Ma M, Han X, Li X, Huang J, Zhao Y, Liu H, Kang J, Kong X, Sun G (2023). Idarubicin-loaded biodegradable microspheres enhance sensitivity to anti-PD1 immunotherapy in transcatheter arterial chemoembolization of hepatocellular carcinoma. Acta Biomater.

[7] Demir T, Lee SS, Kaseb AO, Fisher PB, Sarkar D (2021). Systemic therapy of liver cancer. Mechanisms and therapy of liver cancer.

[8] Holzwanger DJ, Madoff DC (2018). Role of interventional radiology in the management of hepatocellular carcinoma: current status. Chin Clin Oncol.

[9] Chen X-L, Yu H-C, Fan Q-G, Yuan Q, Jiang W-K, Rui S-Z, Zhou W-C (2022). Comparative effectiveness of interventional therapeutic modalities for unresectable hepatocellular carcinoma: a systematic review and network meta-analysis. Oncol Lett.

[10] Liapi E, Geschwind J-FH (2010). Intra-arterial therapies for hepatocellular carcinoma: where do we stand?. Ann Surg Oncol.

[11] Makary MS, Ramsell S, Miller E, Beal EW, Dowell JD (2021). Hepatocellular carcinoma locoregional therapies: outcomes and future horizons. World J Gastroenterol.

[12] Shah RP, Brown KT, Sofocleous CT (2011). Arterially directed therapies for hepatocellular carcinoma. Am J Roentgenol.

[13] Couri T, Pillai A (2019). Goals and targets for personalized therapy for HCC. Hep Intl.

[14] Raoul J-L, Forner A, Bolondi L, Cheung TT, Kloeckner R, de Baere T (2019). Updated use of TACE for hepatocellular carcinoma treatment: how and when to use it based on clinical evidence. Cancer Treat Rev.

[15] Bruix J, Llovet JM (2002). Prognostic prediction and treatment strategy in hepatocellular carcinoma. Hepatology.

[16] Chen Y-P, Zhang J-L, Zou Y, Wu Y-L (2019). Recent advances on polymeric beads or hydrogels as embolization agents for improved transcatheter arterial chemoembolization (TACE). Front Chem.

[17] Llovet JM, Bruix J, Barcelona Clinic Liver Cancer Group (2003). Systematic review of randomized trials for unresectable hepatocellular carcinoma: chemoembolization improves survival. Hepatology.

[18] European Association for the Study of the Liver (2018). EASL clinical practice guidelines: management of hepatocellular carcinoma. J Hepatol.

[19] Chang Y, Jeong SW, Jang JY, Kim YJ (2020). Recent updates of transarterial chemoembolilzation in hepatocellular carcinoma. Int J Mol Sci.

[20] Marelli L, Stigliano R, Triantos C, Senzolo M, Cholongitas E, Davies N, Tibballs J, Meyer T, Patch DW, Burroughs AK (2007). Transarterial therapy for hepatocellular carcinoma: which technique is more effective? A systematic review of cohort and randomized studies. Cardiovasc Intervent Radiol.

[21] Varela M, Real MI, Burrel M, Forner A, Sala M, Brunet M, Ayuso C, Castells L, Montana X, Llovet JM, Bruix J (2007). Chemoembolization of hepatocellular carcinoma with drug eluting beads: efficacy and doxorubicin pharmacokinetics. J Hepatol.

[22] Pesapane F, Nezami N, Patella F, Geschwind JF (2017). New concepts in embolotherapy of HCC. Med Oncol.

[23] Coldwell DM, Stokes KR, Yakes WF (1994). Embolotherapy: agents, clinical applications, and techniques. Radiographics.

[24] Jia G, Van Valkenburgh J, Chen AZ, Chen Q, Li J, Zuo C, Chen K (2022). Recent advances and applications of microspheres and nanoparticles in transarterial chemoembolization for hepatocellular carcinoma. Wiley Interdiscip Rev-Nanomed Nanobiotechnol.

[25] Perez-Lopez A, Martin-Sabroso C, Gomez-Lazaro L, Torres-Suarez AI, Aparicio-Blanco J (2022). Embolization therapy with microspheres for the treatment of liver cancer: state-of-the-art of clinical translation. Acta Biomater.

[26] Ho T-C, Chang C-C, Chan H-P, Chung T-W, Shu C-W, Chuang K-P, Duh T-H, Yang M-H, Tyan Y-C (2022). Hydrogels: properties and applications in biomedicine. Molecules.

[27] Jin S, Wan J, Meng L, Huang X, Guo J, Liu L, Wang C (2015). Biodegradation and toxicity of protease/redox/pH stimuli-responsive PEGlated PMAA nanohydrogels for targeting drug delivery. ACS Appl Mater Interfaces.

[28] Wang C, Xue Y, Lin K, Lu J, Chang J, Sun J (2012). The enhancement of bone regeneration by a combination of osteoconductivity and osteostimulation using beta-CaSiO3/beta-Ca-3(PO4)(2) composite bioceramics. Acta Biomater.

[29] Deng M, Nair LS, Nukavarapu SR, Jiang T, Kanner WA, Li X, Kumbar SG, Weikel AL, Krogman NR, Allcock HR, Laurencin CT (2010). Dipeptide-based polyphosphazene and polyester blends for bone tissue engineering. Biomaterials.

[30] Ko G, Choi JW, Lee N, Kim D, Hyeon T, Kim H-C (2022). Recent progress in liquid embolic agents. Biomaterials.

[31] Lencioni R, de Baere T, Soulen MC, Rilling WS, Geschwind J-FH (2016). Lipiodol transarterial chemoembolization for hepatocellular carcinoma: a systematic review of efficacy and safety data. Hepatology.

[32] Miyayama S, Matsui O, Yamashiro M, Ryu Y, Takata H, Takeda T, Aburano H, Shigenari N (2007). Iodized oil accumulation in the hypovascular tumor portion of early-stage hepatocellular carcinoma after ultraselective transcatheter arterial chemoembolization. Hep Intl.

[33] Chen C-S, Li F-K, Guo C-Y, Xiao J-C, Hu H-T, Cheng H-T, Zheng L, Zong D-W, Ma J-L, Jiang L, Li H-L (2016). Tumor vascularity and lipiodol deposition as early radiological markers for predicting risk of disease progression in patients with unresectable hepatocellular carcinoma after transarterial chemoembolization. Oncotarget.

[34] Wang Q, He Y, Shen M, Huang L, Ding L, Hu J, Dong Y, Fu H, Wang Q, Sun Y (2021). Precision embolism: biocompatible temperature-sensitive hydrogels as novel embolic materials for both mainstream and peripheral vessels. Adv Funct Mater.

[35] Liu M, Wang Y, Chen Y, Li L, Sun Y, Li Y, Yuan Y, Lu P, Zhang W, Pang P (2023). Solvent exchange induced in situ formed hydrogel as liquid embolic agents. Adv Funct Mater.

[36] Zhu J, Marchant RE (2011). Design properties of hydrogel tissue-engineering scaffolds. Expert Rev Med Dev.

[37] Nie J, Pei B, Wang Z, Hu Q (2019). Construction of ordered structure in polysaccharide hydrogel: a review. Carbohydr Polym.

[38] Sun Z, Song C, Wang C, Hu Y, Wu J (2020). Hydrogel-based controlled drug delivery for cancer treatment: a review. Mol Pharm.

[39] Ko G, Choi JW, Shin K, Kim YG, Kang T, Kim D, Lee N, Kim H-C, Hyeon T (2022). In vivo sol-gel reaction of tantalum alkoxide for endovascular embolization. Adv Healthc Mater.

[40] Li H, Qian K, Zhang H, Li L, Yan L, Geng S, Zhao H, Zhang H, Xiong B, Li Z (2021). Pickering gel emulsion of lipiodol stabilized by hairy nanogels for intra-artery embolization antitumor therapy. Chem Eng J.

[41] Go G, Yoo A, Kim Tien N, Nan M, Darmawan BA, Zheng S, Kang B, Kim C-S, Bang D, Lee S (2022). Multifunctional microrobot with real-time visualization and magnetic resonance imaging for chemoembolization therapy of liver cancer. Sci Adv.

[42] Lam M, Reales-Calderon JA, Ow JR, Adriani G, Pavesi A (2021). In vitro 3D liver tumor microenvironment models for immune cell therapy optimization. APL Bioeng.

[43] Gyles DA, Castro LD, Silva JOC, Ribeiro-Costa RM (2017). A review of the designs and prominent biomedical advances of natural and synthetic hydrogel formulations. Eur Polym J.

[44] Ma J, Wang B, Shao H, Zhang S, Chen X, Li F, Liang W (2022). Hydrogels for localized chemotherapy of liver cancer: a possible strategy for improved and safe liver cancer treatment. Drug Deliv.

[45] Hu W, Wang Z, Xiao Y, Zhang S, Wang J (2019). Advances in crosslinking strategies of biomedical hydrogels. Biomater Sci.

[46] Lu L, Yuan S, Wang J, Shen Y, Deng S, Xie L, Yang Q (2018). The formation mechanism of hydrogels. Curr Stem Cell Res Ther.

[47] Yang J, Chen Y, Zhao L, Zhang J, Luo H (2023). Constructions and properties of physically cross-linked hydrogels based on natural polymers. Polym Rev.

[48] Zhang YS, Khademhosseini A (2017). Advances in engineering hydrogels. Science.

[49] Bashir S, Hina M, Iqbal J, Rajpar AH, Mujtaba MA, Alghamdi NA, Wageh S, Ramesh K, Ramesh S (2020). Fundamental concepts of hydrogels: synthesis, properties, and their applications. Polymers.

[50] Wang Y, Jiang W, Li J, Ahommed MS, Wang C, Ji X, Liu Y, Yang G, Ni Y, Lyu G (2023). Zinc-ion engineered plant-based multifunctional hydrogels for flexible wearable strain Sensors, Bio-electrodes and Zinc-ion hybrid capacitors. Chem Eng J.

[51] Pawar SN, Edgar KJ (2012). Alginate derivatization: a review of chemistry, properties and applications. Biomaterials.

[52] Kumar A, Sah DK, Khanna K, Rai Y, Yadav AK, Ansari MS, Bhatt AN (2023). A calcium and zinc composite alginate hydrogel for pre-hospital hemostasis and wound care. Carbohydr Polym.

[53] Bissantz C, Kuhn B, Stahl M (2010). A medicinal chemist’s guide to molecular interactions. J Med Chem.

[54] Li S, Gao Y, Jiang H, Duan L, Gao G (2018). Tough, sticky and remoldable hydrophobic association hydrogel regulated by polysaccharide and sodium dodecyl sulfate as emulsifiers. Carbohyd Polym.

[55] Demott CJ, Jones MR, Chesney CD, Yeisley DJ, Culibrk RA, Hahn MS, Grunlan MA (2022). Ultra-high modulus hydrogels mimicking cartilage of the human body. Macromol Biosci.

[56] Fu L, Li L, Bian Q, Xue B, Jin J, Li J, Cao Y, Jiang Q, Li H (2023). Cartilage-like protein hydrogels engineered via entanglement. Nature.

[57] Khan MJ, Zhang J, Guo Q (2016). Covalent/crystallite cross-linked co-network hydrogels: an efficient and simple strategy for mechanically strong and tough hydrogels. Chem Eng J.

[58] Hassan CM, Peppas NA (2000). Structure and morphology of freeze/thawed PVA hydrogels. Macromolecules.

[59] Muir VG, Burdick JA (2021). Chemically modified biopolymers for the formation of biomedical hydrogels. Chem Rev.

[60] Zhang Z, Fu H, Li Z, Huang J, Xu Z, Lai Y, Qian X, Zhang S (2022). Hydrogel materials for sustainable water resources harvesting & treatment: synthesis, mechanism and applications. Chem Eng J.

[61] Singh B, Pal L (2011). Radiation crosslinking polymerization of sterculia polysaccharide-PVA-PVP for making hydrogel wound dressings. Int J Biol Macromol.

[62] Sahajpal K, Shekhar S, Kumar A, Sharma B, Meena MK, Bhagi AK, Sharma S (2022). Dynamic protein and polypeptide hydrogels based on Schiff base co-assembly for biomedicine. J Mater Chem B.

[63] Mo C, Xiang L, Chen Y (2021). Advances in injectable and self-healing polysaccharide hydrogel based on the Schiff base reaction. Macromol Rapid Commun.

[64] Huang Y, Mu L, Zhao X, Han Y, Guo B (2022). Bacterial growth-induced tobramycin smart release self-healing hydrogel for pseudomonas aeruginosa-infected burn wound healing. ACS Nano.

[65] Mantha S, Pillai S, Khayambashi P, Upadhyay A, Zhang Y, Tao O, Pham HM, Tran SD (2019). Smart hydrogels in tissue engineering and regenerative medicine. Materials.

[66] Fang W-W, Yang G-Y, Fan Z-H, Chen Z-C, Hu X-L, Zhan Z, Hussain I, Lu Y, He T, Tan B-E (2023). Conjugated cross-linked phosphine as broadband light or sunlight-driven photocatalyst for large-scale atom transfer radical polymerization. Nat Commun.

[67] Yu J, Wang K, Fan C, Zhao X, Gao J, Jing W, Zhang X, Li J, Li Y, Yang J, Liu W (2021). An ultrasoft self-fused supramolecular polymer hydrogel for completely preventing postoperative tissue adhesion. Adv Mater.

[68] Meng X, Edgar KJ (2016). “Click” reactions in polysaccharide modification. Prog Polym Sci.

[69] Lueckgen A, Garske DS, Ellinghaus A, Desai RM, Stafford AG, Mooney DJ, Duda GN, Cipitria A (2018). Hydrolytically-degradable click-crosslinked alginate hydrogels. Biomaterials.

[70] Huang Y, Ren J, Qu X (2019). Nanozymes: classification, catalytic mechanisms, activity regulation, and applications. Chem Rev.

[71] Li Z, Lu F, Liu Y (2023). A review of the mechanism, properties, and applications of hydrogels prepared by enzymatic cross-linking. J Agric Food Chem.

[72] Kim M, Kim H, Lee Y-s, Lee S, Kim S-E, Lee U-J, Jung S, Park C-G, Hong J, Doh J (2021). Novel enzymatic cross-linking-based hydrogel nanofilm caging system on pancreatic beta cell spheroid for long-term blood glucose regulation. Sci Adv.

[73] Wei P, Yu X, Fang Y, Wang L, Zhang H, Zhu C, Cai J (2023). Strong and tough cellulose hydrogels via solution annealing and dual cross-linking. Small.

[74] Yuan Y, Shen S, Fan D (2021). A physicochemical double cross-linked multifunctional hydrogel for dynamic burn wound healing: shape adaptability, injectable self-healing property and enhanced adhesion. Biomaterials.

[75] Zhao L, Shi Z, Sun X, Yu Y, Wang X, Wang H, Li T, Zhang H, Zhang X, Wang F (2022). Natural dual-crosslinking bioadhesive hydrogel for corneal regeneration in large-size defects. Adv Healthc Mater.

[76] Gosecka M, Gosecki M, Jaworska-Krych D (2023). Hydrophobized hydrogels: construction strategies, properties, and biomedical applications. Adv Funct Mater.

[77] Narayanaswamy R, Torchilin VP (2019). Hydrogels and their applications in targeted drug delivery. Molecules.

[78] Zhao J, Wang L, Zhang H, Liao B, Li Y (2028). Progress of research in in situ smart hydrogels for local antitumor therapy: a review. Pharmaceutics.

[79] Radu ER, Semenescu A, Voicu SI (2022). Recent advances in stimuli-responsive doxorubicin delivery systems for liver cancer therapy. Polymers.

[80] Zhu J-Q, Wu H, Li Z-L, Xu X-F, Xing H, Wang M-D, Jia H-D, Liang L, Li C, Sun L-Y (2022). Responsive hydrogels based on triggered click reactions for liver cancer. Adv Mater.

[81] Mo C, Luo R, Chen Y (2022). Advances in the stimuli-responsive injectable hydrogel for controlled release of drugs. Macromol Rapid Commun.

[82] Hou S, Wang X, Park S, Jin X, Ma PX (2015). Rapid Self-integrating, injectable hydrogel for tissue complex regeneration. Adv Healthc Mater.

[83] Meng J, Yang X, Huang J, Tuo Z, Hu Y, Liao Z, Tian Y, Deng S, Deng Y, Zhou Z (2023). Ferroptosis-enhanced immunotherapy with an injectable dextran-chitosan hydrogel for the treatment of malignant ascites in hepatocellular carcinoma. Adv Sci.

[84] Zhan J, Wu Y, Wang H, Liu J, Ma Q, Xiao K, Li Z, Li J, Luo F, Tan H (2020). An injectable hydrogel with pH-sensitive and self-healing properties based on 4armPEGDA and N-carboxyethyl chitosan for local treatment of hepatocellular carcinoma. Int J Biol Macromol.

[85] Wang X, Zhang HJ, Yang Y, Chen Y, Zhu X, You X (2023). Biopolymer-based self-healing hydrogels: a short review. Giant.

[86] Qu J, Zhao X, Ma PX, Guo B (2017). pH-responsive self-healing injectable hydrogel based on N-carboxyethyl chitosan for hepatocellular carcinoma therapy. Acta Biomater.

[87] Zeng Z-M, Mo N, Zeng J, Ma F-C, Jiang Y-F, Huang H-S, Liao X-W, Zhu G-Z, Ma J, Peng T (2022). Advances in postoperative adjuvant therapy for primary liver cancer. World J Gastrointest Oncol.

[88] Tang J, Zhang R, Guo M, Shao L, Liu Y, Zhao Y, Zhang S, Wu Y, Chen C (2018). Nucleosome-inspired nanocarrier obtains encapsulation efficiency enhancement and side effects reduction in chemotherapy by using fullerenol assembled with doxorubicin. Biomaterials.

[89] Mittra I, Pal K, Pancholi N, Shaikh A, Rane B, Tidke P, Kirolikar S, Khare NK, Agrawal K, Nagare H, Nair NK (2017). Prevention of chemotherapy toxicity by agents that neutralize or degrade cell-free chromatin. Ann Oncol.

[90] Wolinsky JB, Colson YL, Grinstaff MW (2012). Local drug delivery strategies for cancer treatment: gels, nanoparticles, polymeric films, rods, and wafers. J Control Release.

[91] Majumder P, Baxa U, Walsh STR, Schneider JP (2018). Design of a multicompartment hydrogel that facilitates time-resolved delivery of combination therapy and synergized killing of glioblastoma. Angew Chem-Int Ed.

[92] Kim DY, Kwon DY, Kwon JS, Park JH, Park SH, Oh HJ, Kim JH, Min BH, Park K, Kim MS (2016). Synergistic anti-tumor activity through combinational intratumoral injection of an in-situ injectable drug depot. Biomaterials.

[93] Qi Y, Min H, Mujeeb A, Zhang Y, Han X, Zhao X, Anderson GJ, Zhao Y, Nie G (2018). Injectable hexapeptide hydrogel for localized chemotherapy prevents breast cancer recurrence. ACS Appl Mater Interfaces.

[94] Le Grazie M, Biagini MR, Tarocchi M, Polvani S, Galli A (2017). Chemotherapy for hepatocellular carcinoma: the present and the future. World J Hepatol.

[95] Varela-Lopez A, Battino M, Navarro-Hortal MD, Giampieri F, Forbes-Hernandez TY, Romero-Marquez JM, Collado R, Quiles JL (2019). An update on the mechanisms related to cell death and toxicity of doxorubicin and the protective role of nutrients. Food Chem Toxicol.

[96] Wan J, Geng S, Zhao H, Peng X, Zhou Q, Li H, He M, Zhao Y, Yang X, Xu H (2016). Doxorubicin-induced co-assembling nanomedicines with temperature-sensitive acidic polymer and their in-situ-forming hydrogels for intratumoral administration. J Control Release.

[97] Raudenska M, Balvan J, Fojtu M, Gumulec J, Masarik M (2019). Unexpected therapeutic effects of cisplatin. Metallomics.

[98] Yao X, Panichpisal K, Kurtzman N, Nugent K (2007). Cisplatin nephrotoxicity: a review. Am J Med Sci.

[99] Chen J, Wang D, Wang L-H, Liu W, Chiu A, Shariati K, Liu Q, Wang X, Zhong Z, Webb J (2020). An adhesive hydrogel with “load-sharing” effect as tissue bandages for drug and cell delivery. Adv Mater.

[100] Han Z, Li B, Wang J, Zhang X, Li Z, Dai L, Cao M, Jiang J (2017). Norcantharidin inhibits SK-N-SH neuroblastoma cell growth by induction of autophagy and apoptosis. Technol Cancer Res Treat.

[101] Li X-Y, Guan Q-X, Shang Y-Z, Wang Y-H, Lv S-W, Yang Z-X, Wang R, Feng Y-F, Li W-N, Li Y-J (2021). Metal-organic framework IRMOFs coated with a temperature-sensitive gel delivering norcantharidin to treat liver cancer. World J Gastroenterol.

[102] Zhou Z-L, Yang Y-X, Ding J, Li Y-C, Miao Z-H (2012). Triptolide: structural modifications, structure-activity relationships, bioactivities, clinical development and mechanisms. Nat Prod Rep.

[103] Ling D, Xia H, Park W, Hackett MJ, Song C, Na K, Hui KM, Hyeon T (2014). pH-sensitive nanoformulated triptolide as a targeted therapeutic strategy for hepatocellular carcinoma. ACS Nano.

[104] Zhao X, Liu X, Zhang P, Liu Y, Ran W, Cai Y, Wang J, Zhai Y, Wang G, Ding Y, Li Y (2019). Injectable peptide hydrogel as intraperitoneal triptolide depot for the treatment of orthotopic hepatocellular carcinoma. Acta Pharm Sin B.

[105] Tagde P, Tagde P, Islam F, Tagde S, Shah M, Hussain ZD, Rahman MH, Najda A, Alanazi IS, Germoush MO (2021). The multifaceted role of curcumin in advanced nanocurcumin form in the treatment and management of chronic disorders. Molecules.

[106] Hanafy NAN, Leporatti S, El-Kemary M (2020). Mucoadhesive curcumin crosslinked carboxy methyl cellulose might increase inhibitory efficiency for liver cancer treatment. Mater Sci Eng C-Mater Biol Appl.

[107] Koka K, Verma A, Dwarakanath BS, Papineni RVL (2022). Technological advancements in external beam radiation therapy (EBRT): an indispensable tool for cancer treatment. Cancer Manag Res.

[108] Baskar R, Lee KA, Yeo R, Yeoh K-W (2012). Cancer and radiation therapy: current advances and future directions. Int J Med Sci.

[109] Mohan V, Bruin NM, van de Kamer JB, Sonke JJ, Vogel WV (2021). The increasing potential of nuclear medicine imaging for the evaluation and reduction of normal tissue toxicity from radiation treatments. Eur J Nucl Med Mol Imaging.

[110] Ho S, Lau WY, Leung TW, Johnson PJ (1998). Internal radiation therapy for patients with primary or metastatic hepatic cancer: a review. Cancer.

[111] Lin WY, Tsai SC, Hsieh JF, Wang SJ (2000). Effects of Y-90-microspheres on liver tumors: comparison of intratumoral injection method and intra-arterial injection method. J Nucl Med.

[112] Hwang H, Kim KI, Kwon J, Kim BS, Jeong H-S, Jang SJ, Oh P-S, Park HS, Lim ST, Sohn M-H, Jeong H-J (2017). I-131-labeled chitosan hydrogels for radioembolization: a preclinical study in small animals. Nucl Med Biol.

[113] Kennedy A (2014). Radioembolization of hepatic tumors. J Gastrointest Oncol.

[114] Lee IJ, Seong J (2012). The optimal selection of radiotherapy treatment for hepatocellular carcinoma. Gut Liver.

[115] Karpov T, Postovalova A, Akhmetova D, Muslimov AR, Eletskaya E, V. Zyuzin M, Timin AS,  (2022). Universal chelator-free radiolabeling of organic and inorganic-based nanocarriers with diagnostic and therapeutic isotopes for internal radiotherapy. Chem Mater.

[116] Peng C-L, Shih Y-H, Liang K-S, Chiang P-F, Yeh C-H, Tang IC, Yao C-J, Lee S-Y, Luo T-Y, Shieh M-J (2013). Development of in situ forming thermosensitive hydrogel for radiotherapy combined with chemotherapy in a mouse model of hepatocellular carcinoma. Mol Pharm.

[117] Gudkov SV, Shilyagina NY, Vodeneev VA, Zvyagin AV (2016). Targeted radionuclide therapy of human tumors. Int J Mol Sci.

[118] Lee EJ, Chung HW, Jo J-H, So Y (2019). Radioembolization for the treatment of primary and metastatic liver cancers. Nucl Med Mol Imaging.

[119] Fisher DR (2021). Radiation safety for yttrium-90-polymer composites (RadioGel (TM)) in therapy of solid tumors. Health Phys.

[120] Larson SM, Carrasquillo JA, Cheung N-KV, Press OW (2015). Radioimmunotherapy of human tumours. Nat Rev Cancer.

[121] You J, Zhang R, Xiong C, Zhong M, Melancon M, Gupta S, Nick AM, Sood AK, Li C (2012). Effective photothermal chemotherapy using doxorubicin-loaded gold nanospheres that target EphB4 receptors in tumors. Can Res.

[122] Xi D, Xiao M, Cao J, Zhao L, Xu N, Long S, Fan J, Shao K, Sun W, Yan X, Peng X (2020). NIR light-driving barrier-free group rotation in nanoparticles with an 88.3% photothermal conversion efficiency for photothermal therapy. Adv Mater.

[123] Chen Q, Wang C, Zhan Z, He W, Cheng Z, Li Y, Liu Z (2014). Near-infrared dye bound albumin with separated imaging and therapy wavelength channels for imaging-guided photothermal therapy. Biomaterials.

[124] Zhou Z, Yan Y, Wang L, Zhang Q, Cheng Y (2019). Melanin-like nanoparticles decorated with an autophagy-inducing peptide for efficient targeted photothermal therapy. Biomaterials.

[125] Cheng L, Zhang F, Wang S, Pan X, Han S, Liu S, Ma J, Wang H, Shen H, Liu H, Yuan Q (2019). Activation of prodrugs by NIR-triggered release of exogenous enzymes for locoregional chemo-photothermal therapy. Angew Chem-Int Ed.

[126] Jung HS, Verwilst P, Sharma A, Shin J, Sessler JL, Kim JS (2018). Organic molecule-based photothermal agents: an expanding photothermal therapy universe. Chem Soc Rev.

[127] Dong Q, Wang X, Hu X, Xiao L, Zhang L, Song L, Xu M, Zou Y, Chen L, Chen Z, Tan W (2018). Simultaneous application of photothermal therapy and an anti-inflammatory prodrug using pyrene-aspirin-loaded gold nanorod graphitic nanocapsules. Angew Chem-Int Ed.

[128] Jin R, Yang J, Zhao D, Hou X, Li C, Chen W, Zhao Y, Yin Z, Liu B (2019). Hollow gold nanoshells-incorporated injectable genetically engineered hydrogel for sustained chemo-photothermal therapy of tumor. J Nanobiotechnol.

[129] Siregar S, Oktamuliani S, Saijo Y (2018). A theoretical model of laser heating carbon nanotubes. Nanomaterials.

[130] Liao M-Y, Lai P-S, Yu H-P, Lin H-P, Huang C-C (2012). Innovative ligand-assisted synthesis of NIR-activated iron oxide for cancer theranostics. Chem Commun.

[131] Dang W, Chen W-C, Ju E, Xu Y, Li K, Wang H, Wang K, Lv S, Shao D, Tao Y, Li M (2022). 3D printed hydrogel scaffolds combining glutathione depletion-induced ferroptosis and photothermia-augmented chemodynamic therapy for efficiently inhibiting postoperative tumor recurrence. J Nanobiotechnol.

[132] Huang S, Ma Z, Sun C, Zhou Q, Li Z, Wang S, Yan Q, Liu C, Hou B, Zhang C (2022). An injectable thermosensitive hydrogel loaded with a theranostic nanoprobe for synergistic chemo-photothermal therapy for multidrug-resistant hepatocellular carcinoma. J Mater Chem B.

[133] Cai W, Gao H, Chu C, Wang X, Wang J, Zhang P, Lin G, Li W, Liu G, Chen X (2017). Engineering phototheranostic nanoscale metal-organic frameworks for multimodal imaging-guided cancer therapy. ACS Appl Mater Interfaces.

[134] Deng K, Hou Z, Deng X, Yang P, Li C, Lin J (2015). Enhanced antitumor efficacy by 808 nm laser-induced synergistic photothermal and photodynamic therapy based on a indocyanine-green-attached W18O49 nanostructure. Adv Funct Mater.

[135] Sohretoglu D, Huang S (2018). *Ganoderma lucidum* polysaccharides as an anti-cancer agent. Anticancer Agents Med Chem.

[136] Xia Q-H, Lu C-T, Tong M-Q, Yue M, Chen R, Zhuge D-L, Yao Q, Xu H-L, Zhao Y-Z (2021). *Ganoderma lucidum* polysaccharides enhance the abscopal effect of photothermal therapy in hepatoma-bearing mice through immunomodulatory, anti-proliferative, pro-apoptotic and anti-angiogenic. Front Pharmacol.

[137] Noh S-H, Moon SH, Shin T-H, Lim Y, Cheon J (2017). Recent advances of magneto-thermal capabilities of nanoparticles: from design principles to biomedical applications. Nano Today.

[138] Hauser AK, Wydra RJ, Stocke NA, Anderson KW, Hilt JZ (2015). Magnetic nanoparticles and nanocomposites for remote controlled therapies. J Control Release.

[139] Johannsen M, Gneueckow U, Thiesen B, Taymoorian K, Cho CH, Waldofner N, Scholz R, Jordan A, Loening SA, Wust P (2007). Thermotherapy of prostate cancer using magnetic nanoparticles: feasibility, imaging, and three-dimensional temperature distribution. Eur Urol.

[140] van Landeghem FKH, Maier-Hauff K, Jordan A, Hoffmann K-T, Gneveckow U, Scholz R, Thiesen B, Brueck W, von Deimling A (2009). Post-mortem studies in glioblastoma patients treated with thermotherapy using magnetic nanoparticles. Biomaterials.

[141] Mueller S (2009). Magnetic fluid hyperthermia therapy for malignant brain tumors-an ethical discussion. Nanomed-Nanotechnol Biol Med.

[142] Pan J, Xu Y, Wu Q, Hu P, Shi J (2021). Mild magnetic hyperthermia-activated innate immunity for liver cancer therapy. J Am Chem Soc.

[143] Qian K-Y, Song Y, Yan X, Dong L, Xue J, Xu Y, Wang B, Cao B, Hou Q, Peng W (2020). Injectable ferrimagnetic silk fibroin hydrogel for magnetic hyperthermia ablation of deep tumor. Biomaterials.

[144] Chen S, Song Y, Yan X, Dong L, Xu Y, Xuan S, Shu Q, Cao B, Hu J, Xing H (2022). Injectable magnetic montmorillonite colloidal gel for the postoperative treatment of hepatocellular carcinoma. J Nanobiotechnol.

[145] Yan X, Sun T, Song Y, Peng W, Xu Y, Luo G, Li M, Chen S, Fang W-W, Dong L (2022). In situ thermal-responsive magnetic hydrogel for multidisciplinary therapy of hepatocellular carcinoma. Nano Lett.

[146] Huang W, Ling S, Li C, Omenetto FG, Kaplan DL (2018). Silkworm silk-based materials and devices generated using bio-nanotechnology. Chem Soc Rev.

[147] Wang Y, Guo J, Zhou L, Ye C, Omenetto FG, Kaplan DL, Ling S (2018). Design, fabrication, and function of silk-based nanomaterials. Adv Funct Mater.

[148] Seib FP, Pritchard EM, Kaplan DL (2013). Self-assembling doxorubicin silk hydrogels for the focal treatment of primary breast cancer. Adv Funct Mater.

[149] Ribeiro VP, Silva-Correia J, Goncalves C, Pina S, Radhouani H, Montonen T, Hyttinen J, Roy A, Oliveira AL, Reis RL, Oliveira JM (2018). Rapidly responsive silk fibroin hydrogels as an artificial matrix for the programmed tumor cells death. PLoS ONE.

[150] Zhang D, Chu Y, Qian H, Qian L, Shao J, Xu Q, Yu L, Li R, Zhang Q, Wu F (2020). Antitumor activity of thermosensitive hydrogels packaging gambogic acid nanoparticles and tumor-penetrating peptide iRGD against gastric cancer. Int J Nanomed.

[151] Huan L, Liang L-H, He X-H (2016). Role of microRNAs in inflammation-associated liver cancer. Cancer Biol Med.

[152] Edmondson HA, Peters RL (1983). Tumors of the liver: pathologic features. Semin Roentgenol.

[153] Llovet JM, Castet F, Heikenwalder M, Maini MK, Mazzaferro V, Pinato DJ, Pikarsky E, Zhu AX, Finn RS (2022). Immunotherapies for hepatocellular carcinoma. Nat Rev Clin Oncol.

[154] Kirchhammer N, Trefny MP, Maur PAD, Laubli H, Zippelius A (2022). Combination cancer immunotherapies: emerging treatment strategies adapted to the tumor microenvironment. Sci Transl Med.

[155] Zhao Q, Wang Y, Zhao B, Chen H, Cai Z, Zheng Y, Zeng Y, Zhang D, Liu X (2022). Neoantigen immunotherapeutic-gel combined with TIM-3 blockade effectively restrains orthotopic hepatocellular carcinoma progression. Nano Lett.

[156] Hu Y, Chen L, Liu M, Ma Z, Zhou C, Yao Z, Zhang S, Song C, Wang Z, Zhu X (2023). Multifunctional immunotherapeutic gel prevented postoperative recurrence of hepatocellular carcinoma. Chem Eng J.

[157] Shi D, Zhang H, Zhang H, Li L, Li S, Zhao Y, Zheng C, Nie G, Yang X (2022). The synergistic blood-vessel-embolization of coagulation fusion protein with temperature sensitive nanogels in interventional therapies on hepatocellular carcinoma. Chem Eng J.

[158] Liu Y, Shi D, Ren Y, Li L, Zhao Y, Zheng C, Yang X (2022). The immune-chemo-embolization effect of temperature sensitive gold nanomedicines against liver cancer. Nano Res.

[159] van der Pol CB, Lim CS, Sirlin CB, McGrath TA, Salameh J-P, Bashir MR, Tang A, Singal AG, Costa AF, Fowler K, McInnes MDF (2019). Accuracy of the liver imaging reporting and data system in computed tomography and magnetic resonance image analysis of hepatocellular carcinoma or overall malignancy-a systematic review. Gastroenterology.

[160] Marrero JA, Kulik LM, Sirlin CB, Zhu AX, Finn RS, Abecassis MM, Roberts LR, Heimbach JK (2018). Diagnosis, staging, and management of hepatocellular carcinoma: 2018 practice guidance by the American Association for the study of liver diseases. Hepatology.

[161] Sitti M, Ceylan H, Hu W, Giltinan J, Turan M, Yim S, Diller E (2015). Biomedical applications of untethered mobile milli/microrobots. Proc IEEE.

[162] Aziz A, Pane S, Iacovacci V, Koukourakis N, Czarske J, Menciassi A, Medina-Sanchez M, Schmidt OG (2020). Medical imaging of microrobots: toward in vivo applications. ACS Nano.

[163] Landsman ML, Kwant G, Mook GA, Zijlstra WG (1976). Light-absorbing properties, stability, and spectral stabilization of indocyanine green. J Appl Physiol.

[164] Kokudo N, Ishizawa T (2012). Clinical application of fluorescence imaging of liver cancer using indocyanine green. Liver Cancer.

[165] Gotoh K, Yamada T, Ishikawa O, Takahashi H, Eguchi H, Yano M, Ohigashi H, Tomita Y, Miyamoto Y, Imaoka S (2009). HOW I DO IT a novel image-guided surgery of hepatocellular carcinoma by indocyanine green fluorescence imaging navigation. J Surg Oncol.

[166] Ishizawa T, Fukushima N, Shibahara J, Masuda K, Tamura S, Aoki T, Hasegawa K, Beck Y, Fukayama M, Kokudo N (2009). Real-time identification of liver cancers by using indocyanine green fluorescent imaging. Cancer.

[167] Salis A, Rassu G, Budai-Szucs M, Benzoni I, Csanyi E, Berko S, Maestri M, Dionigi P, Porcu EP, Gavini E, Giunchedi P (2015). Development of thermosensitive chitosan/glicerophospate injectable in situ gelling solutions for potential application in intraoperative fluorescence imaging and local therapy of hepatocellular carcinoma: a preliminary study. Expert Opin Drug Deliv.

[168] Idee J-M, Guiu B (2013). Use of Lipiodol as a drug-delivery system for transcatheter arterial chemoembolization of hepatocellular carcinoma: a review. Crit Rev Oncol Hematol.

[169] Oh MH, Lee N, Kim H, Park SP, Piao Y, Lee J, Jun SW, Moon WK, Choi SH, Hyeon T (2011). Large-scale synthesis of bioinert tantalum oxide nanoparticles for X-ray computed tomography imaging and bimodal image-guided sentinel lymph node mapping. J Am Chem Soc.

[170] Shin K, Choi JW, Ko G, Baik S, Kim D, Park OK, Lee K, Cho HR, Han SI, Lee SH (2017). Multifunctional nanoparticles as a tissue adhesive and an injectable marker for image-guided procedures. Nat Commun.

[171] Mohandas G, Oskolkov N, McMahon MT, Walczak P, Janowski M (2014). Porous tantalum and tantalum oxide nanoparticles for regenerative medicine. Acta Neurobiol Exp.

[172] Lee KX, Shameli K, Yew YP, Teow S-Y, Jahangirian H, Rafiee-Moghaddam R, Webster TJ (2020). Recent developments in the facile bio-synthesis of gold nanoparticles (AuNPs) and their biomedical applications. Int J Nanomed.

[173] Cui D, Jiang G, Luo F (2014). Surface-modified gold nanoparticles for delivery applications. J Shenyang Pharm Univ.

[174] Lei X, Shao C, Shou X, Shi K, Shi L, Zhao Y (2021). Porous hydrogel arrays for hepatoma cell spheroid formation and drug resistance investigation. Bio-Des Manuf.

[175] Alley MC, Scudiero DA, Monks A, Hursey ML, Czerwinski MJ, Fine DL, Abbott BJ, Mayo JG, Shoemaker RH, Boyd MR (1988). Feasibility of drug screening with panels of human tumor cell lines using a microculture tetrazolium assay. Can Res.

[176] Asghar W, El Assal R, Shafiee H, Pitteri S, Paulmurugan R, Demirci U (2015). Engineering cancer microenvironments for in vitro 3-D tumor models. Mater Today.

[177] Edmondson R, Broglie JJ, Adcock AF, Yang L (2014). Three-dimensional cell culture systems and their applications in drug discovery and cell-based biosensors. Assay Drug Dev Technol.

[178] de Aberasturi DJ, Henriksen-Lacey M, Litti L, Langer J, Liz-Marzan LM (2020). Using SERS tags to image the three-dimensional structure of complex cell models. Adv Funct Mater.

[179] Shao C, Liu Y, Chi J, Chen Z, Wang J, Zhao Y (2019). Droplet microarray on patterned butterfly wing surfaces for cell spheroid culture. Langmuir.

[180] Guo W, Yang K, Qin X, Luo R, Wang H, Huang R (2022). Polyhydroxyalkanoates in tissue repair and regeneration. Eng Regen.

[181] Shao C, Zhang Q, Kuang G, Fan Q, Ye F (2022). Construction and application of liver cancer models in vitro. Eng Regen.

[182] Henke E, Nandigama R, Ergun S (2020). Extracellular matrix in the tumor microenvironment and its impact on cancer therapy. Front Mol Biosci.

[183] Lou J, Mooney DJ (2022). Chemical strategies to engineer hydrogels for cell culture. Nat Rev Chem.

[184] Guo Y, Bae J, Fang Z, Li P, Zhao F, Yu G (2020). Hydrogels and hydrogel-derived materials for energy and water sustainability. Chem Rev.

[185] Chen H, Wei X, Chen H, Wei H, Wang Y, Nan W, Zhang Q, Wen X (2019). The study of establishment of an in vivo tumor model by three-dimensional cells culture systems methods and evaluation of antitumor effect of biotin-conjugated pullulan acetate nanoparticles. Artif Cells Nanomed Biotechnol.

[186] Wu X, Huang W, Wu W-H, Xue B, Xiang D, Li Y, Qin M, Sun F, Wang W, Zhang W-B, Cao Y (2018). Reversible hydrogels with tunable mechanical properties for optically controlling cell migration. Nano Res.

[187] Butcher DT, Alliston T, Weaver VM (2009). A tense situation: forcing tumour progression. Nat Rev Cancer.

[188] Liang Y, Jeong J, DeVolder RJ, Cha C, Wang F, Tong YW, Kong H (2011). A cell-instructive hydrogel to regulate malignancy of 3D tumor spheroids with matrix rigidity. Biomaterials.

[189] Kim C-H, Suhito IR, Angeline N, Han Y, Son H, Luo Z, Kim T-H (2020). Vertically coated graphene oxide micro-well arrays for highly efficient cancer spheroid formation and drug screening. Adv Healthc Mater.

[190] Zhang L, Xiang Y, Zhang H, Cheng L, Mao X, An N, Zhang L, Zhou J, Deng L, Zhang Y (2020). A biomimetic 3D-self-forming approach for microvascular scaffolds. Adv Sci.

[191] Lee S-Y, Teng Y, Son M, Ku B, Hwang HJ, Tergaonkar V, Chow PK-H, Lee DW, Nam D-H (2021). Three-dimensional aggregated spheroid model of hepatocellular carcinoma using a 96-pillar/well plate. Molecules.

[192] van de Wetering M, Francies HE, Francis JM, Bounova G, Iorio F, Pronk A, van Houdt W, van Gorp J, Taylor-Weiner A, Kester L (2015). Prospective derivation of a living organoid biobank of colorectal cancer patients. Cell.

[193] Fong ELS, Toh TB, Lin X, Liu Z, Hooi L, Rashid MBMA, Benoukraf T, Chow EK-H, Huynh TH, Yu H (2018). Generation of matched patient-derived xenograft in vitro-in vivo models using 3D macroporous hydrogels for the study of liver cancer. Biomaterials.

[194] Zhu L, Shao C, Chen H, Chen Z, Zhao Y (2021). Hierarchical hydrogels with ordered micro-nano structures for cancer-on-a-chip construction. Research.

